# A Bayesian Implementation of the Multispecies Coalescent Model with Introgression for Phylogenomic Analysis

**DOI:** 10.1093/molbev/msz296

**Published:** 2019-12-06

**Authors:** Tomáš Flouri, Xiyun Jiao, Bruce Rannala, Ziheng Yang

**Affiliations:** Department of Genetics, Evolution and Environment, University College London, London, United Kingdom; Department of Genetics, Evolution and Environment, University College London, London, United Kingdom; Department of Evolution and Ecology, University of California, Davis, Davis, CA; Department of Genetics, Evolution and Environment, University College London, London, United Kingdom

**Keywords:** Bayesian inference, Bpp, introgression, multispecies coalescent with introgression, MSci, MCMC

## Abstract

Recent analyses suggest that cross-species gene flow or introgression is common in nature, especially during species divergences. Genomic sequence data can be used to infer introgression events and to estimate the timing and intensity of introgression, providing an important means to advance our understanding of the role of gene flow in speciation. Here, we implement the multispecies-coalescent-with-introgression model, an extension of the multispecies-coalescent model to incorporate introgression, in our Bayesian Markov chain Monte Carlo program Bpp. The multispecies-coalescent-with-introgression model accommodates deep coalescence (or incomplete lineage sorting) and introgression and provides a natural framework for inference using genomic sequence data. Computer simulation confirms the good statistical properties of the method, although hundreds or thousands of loci are typically needed to estimate introgression probabilities reliably. Reanalysis of data sets from the purple cone spruce confirms the hypothesis of homoploid hybrid speciation. We estimated the introgression probability using the genomic sequence data from six mosquito species in the *Anopheles gambiae* species complex, which varies considerably across the genome, likely driven by differential selection against introgressed alleles.

## Introduction

A number of recent studies have revealed cross-species hybridization/introgression in a variety of species ranging from *Arabidopsis* (Arnold et al. 2016), butterflies (Martin et al. 2013), corals (Mao et al. 2018), and birds (Ellegren et al. 2012) to mammals such as bears (Liu et al. 2014; Kumar et al. 2017), cattle (Wu et al. 2018), gibbons (Chan et al. 2013; Shi and Yang 2018), and hominins (Nielsen et al. 2017). Introgression may play an important role in speciation (Harrison and Larson 2014; Mallet et al. 2016; Martin and Jiggins 2017). Inference of introgression and estimation of migration rates can contribute to our understanding of the speciation process (Mallet et al. 2016; Martin and Jiggins 2017). Furthermore, introgression and deep coalescence (or incomplete lineage sorting) are two major challenges for species tree reconstruction (Martin et al. 2013; Liu et al. 2014; Fontaine et al. 2015).

There is a large body of literature on the use of networks to model non-treelike evolution (Huson et al. 2011) and a number of methods have been developed to detect cross-species gene flow. Most use summaries of the multilocus sequence data such as the estimated gene trees (Solis-Lemus and Ane 2016; Wen et al. 2016; Solis-Lemus et al. 2017; Cao et al. 2019) or the counts of parsimony-informative site patterns (Green et al. 2010; Durand et al. 2011; Blischak et al. 2018). See Degnan (2018) and Folk et al. (2018) for recent reviews. We focus on coalescent-based full-likelihood models applied to multilocus sequence alignments from closely related species. These come in two forms. The isolation-with-migration (IM) model assumes continuous migration, with species exchanging migrants at certain rates every generation (Hey and Nielsen 2004; Hey 2010), whereas the multispecies-coalescent-with-introgression (MSci) model assumes episodic introgression/hybridization (Yu et al. 2014). Although the probability density of the gene trees under the IM (Hey 2010) and MSci (Yu et al. 2014) models is straightforward to compute, developing a Bayesian Markov chain Monte Carlo (MCMC) program that is feasible for use with genome-scale data sets has been challenging. The space of unknown genealogical histories (including the migration/introgression histories) is large, and constraints between the species tree and the gene trees make it difficult to traverse the parameter space in the posterior. Current implementations of full-likelihood methods through MCMC include IMa3 (Hey 2010; Hey et al. 2018) for the IM model, and *beast (Zhang et al. 2018; Jones 2019) and PHyloNEt/mcmc-seq (Wen and Nakhleh 2018; Wen et al. 2018) for the MSci model. It does not appear computationally feasible to apply those programs to realistically sized data sets, with more than 200 loci, say.

In this article, we extend the multispecies-coalescent (MSC) model in the Bpp program (Rannala and Yang 2003; Burgess and Yang 2008; Yang 2015) to accommodate introgression, resulting in the MSci model (Degnan 2018). The MSci model can be used to estimate species divergence times and the number, timings, and intensities of introgression events. By accommodating gene flow and providing more reliable estimates of evolutionary parameters, the model may also be used in heuristic species delimitation (Jackson et al. 2017; Leaché et al. 2019). We conduct simulation to examine the statistical properties of the method, in comparison with two summary methods, SNaQ (Solis-Lemus and Ane 2016; Solis-Lemus et al. 2017) and Hyde (Blischak et al. 2018). We apply the new method to data sets of purple cone spruce (Sun et al. 2014; Zhang et al. 2018), budding yeast (Rokas et al. 2003; Wen and Nakhleh 2018), and *Anopheles* mosquito genomes (Fontaine et al. 2015; Thawornwattana et al. 2018a), to examine the computational efficiency of our algorithms in comparison with previous implementations (Wen and Nakhleh 2018; Zhang et al. 2018) and to estimate the introgression probability and to study its variation across the genome.

## Results

### The MSci Model

**FIg. 1.**
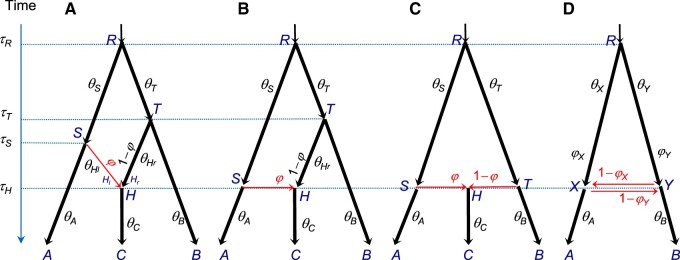
The MSci model with four different types of hybridization (introgression or admixture) events. In (*A*), two parental species *SH* and *TH* merge to form a hybrid species *H*, at time *τ_H_*, leading to extinction of the parental species. In (*B*), there is introgression from species *RSA* to species *THC* at time $\tau_{H}=\tau_{S}$, with introgression probability φ. In (*C*), species *RSA* and *RTB* come into contact to form hybrid species *H* at time $\tau_{S}=\tau_{H}=\tau_{T}$, which evolves into species *C*, while the two parent species become *A* and *B*. In (*D*), bidirectional introgression occurs between species *RXA* and *RYB* at time $\tau_{X}=\tau_{Y}$, with introgression probabilities $\varphi_{X}$ and $\varphi_{Y}$. Parameters in the model include speciation/hybridization times (*τ*s), population sizes (*θ*s), and introgression probabilities (φs). The models are represented using the extended Newick notation (see Appendix) (Cardona et al. 2008), as (*A*–*C*): ((A,(C)H)S,(H,B)T)R and (*D*): ((A,Y)X,(X,B)Y)R. Arrows indicate the direction of time from parent to child or from source to target populations.

We extend the MSC model (Rannala and Yang 2003) to accommodate cross-species hybridization (or introgression) by introducing hybridization (or *H*) nodes (fig. 1). Each *H* node has two parents (*H*_l_ and *H*_r_, for left and right) and one daughter, although the *H* node and its parents may have the same age when there is an admixture or horizontal gene transfer (fig. 1*B* and *C*). In model A, both parental species become extinct after hybridization, whereas model B represents an introgression from species *RSA* into *THC*. Model C represents hybrid speciation, whereas model D represents bidirectional introgression (Kubatko 2009).

When we trace a lineage backward in time and reach an *H* event, the lineage may traverse either the left or the right parental species, according to the introgression probability (φ or 1−φ). This probability is equivalent to the “inheritance probability” *γ* of Yu et al. (2014) and the “heritability” of Solis-Lemus and Ane (2016). The MSci model includes three sets of parameters: the speciation and introgression times (τ); the population size parameters (θ), with each θ=4Nμ, where *N* is the effective population size and *μ* is the mutation rate per generation per site; and the introgression probabilities (ϕ). Both *τ*s and *θ*s are measured by the expected number of mutations per site. Here, we assume that the MSci model is fixed; cross-model MCMC moves will be developed in future work.

Let $G=\{G_{i}\}$ be the set of gene trees for the *L* loci. For each locus *i*, *G_i_* represents the gene-tree topology, the branch lengths (coalescent times), and the paths taken at the *H* nodes, indicated by a set of flags for each gene-tree branch, with l for left, r for right and ∅ for null (meaning that the branch does not pass the *H* node). The data $X=\{X_{i}\}$ are the sequence alignments at the *L* loci. Sites within the same locus are assumed to share the same genealogical history, whereas the gene trees and coalescent times are assumed to be independent among loci given the species tree and parameters. The ideal data for this kind of analysis are loosely linked short genomic segments (called loci), so that recombination within a locus is unimportant, whereas different loci are largely independent (Burgess and Yang 2008; Lohse et al. 2016; Hey et al. 2018). The Bayesian formulation consists of two components. The first is the probability density of gene trees given the species tree under the MSci model, $f(G_{i}|\tau ,\theta ,\phi )$, given in Yu et al. (2014) although here *G_i_* includes the flags for hybrid nodes. Note that this differs from the density given by Kubatko (2009), as pointed out by Solis-Lemus and Ane (2016). The second component is the likelihood of the sequence data at each locus *i* given the gene tree, $f(X_{i}|G_{i})$ (Felsenstein 1981). The posterior probability density of the parameters on the species tree given sequence data is then

(1) $$f(\tau ,\theta ,\phi |X)\propto f(\tau ,\theta ,\phi )\prod_{i=1}^{L}\int_{G_{i}}f(G_{i}|\tau ,\theta ,\phi )f(X_{i}|G_{i})\;dG_{i},$$

where f(τ,θ,ϕ) is the prior on parameters. We assign inverse-gamma priors on *θ*s and *τ*s and a beta prior on φ.

**FIg. 2.**
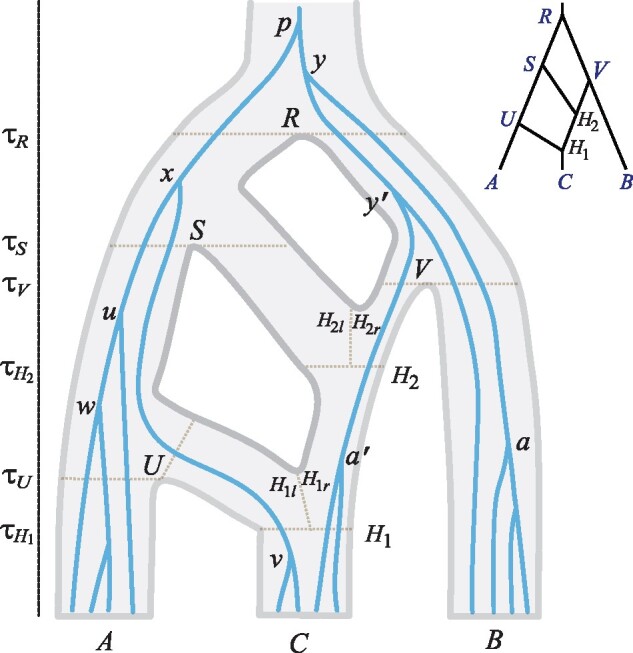
A species tree for three species (*A*, *B*, *C*) with a gene tree for 12 sequences running inside it to illustrate the gene-tree node-age move and the gene-tree SPR move. There are four speciation nodes (R,S,U,V) and two hybridization nodes (*H*_1_ and *H*_2_). This MSci model is also used in simulation, where the model is referred to as “2H”.

We have implemented six MCMC proposals to average over the gene trees (*G_i_*) and sample from the posterior (eq. 1). Those proposals 1) change node ages on gene trees, 2) change gene-tree topologies using subtree pruning and regrafting (SPR), 3) change *θ*s on the species tree using sliding windows, 4) change *τ*s on the species tree using a variant of the rubber-band algorithm (Rannala and Yang 2003), 5) changing all node ages on the species tree and gene trees using a multiplier, and 6) change the introgression probabilities φs using sliding windows. The proposals are detailed in Materials and Methods using the example species tree model of figure 2.

### Simulation Study

We conducted three sets of simulation to examine the performance of Bpp in different situations.

The first set includes multiple sequences from each species and examines Bpp estimation of parameters in the MSci model and the impact of factors such as the number of loci, the introgression probability φ, and the species tree model. We used models A and C of figure 1. Each data set consisted of 10, 100, or 1,000 loci, with 10 sequences from each species per locus (and 30 sequences in total). We used two values for φ (0.1 and 0.5) and two values for *θ* (0.001 and 0.01). Either a JC (Jukes and Cantor 1969) or a GTR + Γ (Yang 1994a, 1994b) substitution model was used to simulate data, but JC was always used to analyze them. The results may be summarized as follows (supplementary figs. S1–S8, Supplementary Material online).

- First, there were large variations in estimation precision and accuracy among the different parameters. For example, estimates of *θ*s for modern species were accurate even in small data sets in all combinations of trees, models, and *θ* values. In contrast, *θ*s for some ancestral species (such as *θ_S_*, *θ_T_*, $\theta_{H_{l}}$, and $\theta_{H_{r}}$ in model A when the true θ=0.001) were poorly estimated, with the posterior dominated by the prior even with 100 or 1,000 loci. Those parameters were hard to estimate as very few sequences enter or coalesce in the populations. These same parameters were much better estimated when the true θ=0.01 as then many more sequences could enter and coalesce in the ancestral species. For similar reasons, the ages of ancestral nodes such as *τ_H_* and *τ_S_* were much better estimated when the true θ=0.01 than when θ=0.001.
- Second, parameter estimates under model C were more precise than under model A, because the former has 9 parameters, whereas the latter 13.
- Third, there were virtually no differences in the results whether the data were simulated under JC or GTR + Γ. As the role of the mutation model in Bpp is to correct for multiple hits at the same site and as the simulated sequences are highly similar, the choice of the mutation model is unimportant. Similar observations were made in previous simulations examining species tree estimation without introgression (Shi and Yang 2018).
- Last, the data size (the number of loci) had a huge impact on the precision and accuracy of estimation. In particular, data of only 10 or 100 loci did not produce reliable estimates of φ, whereas estimates from 1,000 loci were both precise (with narrow intervals) and accurate (close to the true values). Because the MSci models are parameter rich, large data sets in the order of 1,000 loci are necessary for reliable inference.

In the second set of simulations we compared Bpp with two summary methods: SNaQ (Solis-Lemus and Ane 2016; Solis-Lemus et al. 2017) and Hyde (Blischak et al. 2018), using one sequence per species. We simulated data under model A, with three ingroup species (*A*, *B*, and *C*), as well as two outgroup species *D* and *E*, as required by SNaQ (Solis-Lemus et al. 2017). One sequence was sampled per species per locus. The data were then analyzed using the three programs to estimate φ (supplementary fig. S9, Supplementary Material online). Data size had a large impact on the precision and accuracy of the estimates. All three methods performed poorly with 10 or 100 loci (or gene trees), but the estimates were close to the true values with 1,000 loci. Overall, the three methods had similar performance in estimating φ. In some small data sets, SNaQ and Hyde had extreme estimates of 0, whereas Bpp always produced nonzero estimates, due to Bayesian shrinkage through the prior.

Note that the problem examined here is a conventional parameter estimation problem under a well-specified model, so that standard statistical theory applies, which states that the Bayesian method has optimal large-sample properties (O’Hagan and Forster 2004). The small differences among the methods suggest that information in the data concerning φ mostly lies in the proportions of gene trees, which may be reliably estimated even if phylogenetic information content at each individual locus is low. We note that Bpp has several advantages. 1) Bpp accommodates the uncertainties in the data appropriately and produces posterior credible intervals (CIs), whereas SNaQ and Hyde generate point estimates only. 2) Bpp estimates all 13 parameters in the model, whereas SNaQ and Hyde estimate only 2 (φ and the internal branch length) with the others unidentifiable. Estimates of ancestral population sizes (*θ*s) and species divergence and introgression times (*τ*s) may be useful for understanding the evolutionary history of the species. 3) Bpp can use loci of any data configuration, including loci with sequences from only one or two species, which are informative for Bpp but carry no information about gene trees. 4) Some introgression models or biologically important scenarios are unidentifiable using SNaQ and Hyde but can be analyzed using Bpp (see below). In contrast, SNaQ and Hyde have a huge computational advantage over Bpp and may be very useful for exploratory analysis in large data sets.

The third set of simulations explored the performance of Bpp under models that are unidentifiable using SNaQ and Hyde. We used model D of figure 1 and model 2H of figure 2, with results in supplementary figure S10, Supplementary Material online. Model D represents bidirectional introgression between two species. Population size parameters (*θ*s) for modern species *A* and *B* were well estimated even with 100 loci, as was *θ_R_* for the root, but *θ_X_* for species *X* (branch *X*-*R*) and *θ_Y_* (for branch *Y*-*R*) were more poorly estimated (supplementary fig. S10*A*, Supplementary Material online). Both *τ* parameters were well estimated. The introgression probabilities $\varphi_{X}$ and $\varphi_{Y}$ were poorly estimated in small data sets of 10 or 100 loci but were fairly accurate with 1,000 loci.

Model 2H (fig. 2) involves two introgression events on a species tree of three species. There were large differences in information content for different parameters (supplementary fig. S10*B*, Supplementary Material online). Parameters *θ*s for modern species were well estimated even in small data sets, but *θ*s for most ancestral species were poorly estimated because of lack of coalescent events in those populations. Parameter $\theta_{H_{1r}}$ was more accurately estimated than $\theta_{H_{1l}}$ because more sequences passed node *H*_1_ from the right (with probability 1−φ=0.9) than from the left (with φ=0.1), and $\theta_{H_{1l}}$ and $\theta_{H_{1r}}$ were more reliably estimated than $\theta_{H_{2l}}$ and $\theta_{H_{2r}}$ because more sequences passed node *H*_1_ than node *H*_2_. Similarly $\tau_{H_{1}}$ was better estimated than $\tau_{H_{2}}$. With 1,000 loci, all six node ages (for $R,S,U,V,H_{1}$, and *H*_2_) were well estimated. The two introgression probabilities ($\varphi_{H_{1}}$ and $\varphi_{H_{2}}$) were poorly estimated with 10 or 100 loci but were reliably estimated when 1,000 loci were used.

In summary, in both introgression scenarios of models D and 2H, where SNaQ and Hyde are inapplicable, Bpp appears to be a well-behaved method, providing reliable estimates of introgression probabilities as well as species divergence and introgression times.

### Analysis of the Purple Cone Spruce Data

**FIg. 3.**
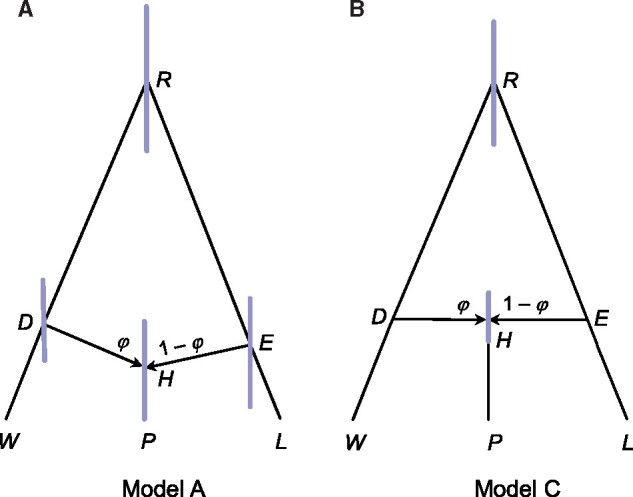
Two species trees for the purple cone spruce *Picea purpurea* (P) from the Qinghai–Tibet Plateau, and two parental species *P. wilsonii* (W) and *P. likiangensis* (L). These correspond to (A) Model A and (B) Model C of figure 1. The branch lengths represent the posterior means of divergence times (*τ*s) estimated from the “Full” data set, with node bars showing the 95% HPD intervals (see supplementary table S1, Supplementary Material online).

We analyzed three data sets concerning the origin of the purple cone spruce in the Qinghai–Tibet Plateau, *Picea purpurea*, hypothesized to be a hybrid species, formed through homoploid hybridization between *P. wilsonii* (*W*) and *P. likiangensis* (*L*) (Sun et al. 2014). Two small data sets were previously analyzed using *beast under model A of figure 3, whereas the third one (the “Full” data) is a much larger data set from which the first two were sampled. We attempted to apply PHyloNEt/mcmc-seq (Wen and Nakhleh 2018) to analyze any of those data sets but were unsuccessful. The program used all 144 cores on our server and did not produce any output after 5 days. The data sets appeared to be too large for the program.

Parameter estimates under model A from the two small data sets were similar to those in Zhang et al. (2018), with φ estimates between 0.32 and 0.44, although the estimates involved large uncertainties (supplementary table S1, Supplementary Material online). The uncertainty is apparently due to the use of only 11 loci (although many sequences are available at each locus) and the shallowness of the species tree, with species divergence times being comparable with coalescent times (or with similar *τ*s and *θ*s). Accommodating rate variation among loci had very small effects. The full data produced similar parameter estimates to the two small data sets, but φ is larger, at 0.47–0.49. We also applied model C (fig. 3) to the full data, which produced more precise estimates because of the smaller number of parameters (fig. 3 and supplementary table S1, Supplementary Material online). The φ estimate under model C was 0.53, with the 95% highest posterior density (HPD) CI to be 0.36–0.71.

Note that the models represent different biological scenarios. Model A assumes the existence, and subsequent extinction at the time of hybridization, of species *DH* and *EH* (fig. 3). This is not a very plausible model (Sun et al. 2014). Model C represents speciation through homoploid hybridization, with species *RDW* and *REL* coming into contact and forming a hybrid species (*H*) at time *τ_H_*. A possible scenario is that changes in species distribution may have led to habitat overlap between *P. wilsonii* and *P. likiangensis* during the Quaternary glaciation in the central Qinghai–Tibet Plateau (Sun et al. 2014). We calculated marginal likelihoods (Bayes factors) to compare models A–C (fig. 1). The log marginal likelihood was –18,361 for model A, –18,359 and –18,361 for two cases of model B (with $\tau_{H}=\tau_{D}$ and $\tau_{H}=\tau_{E}$, respectively), and –18,362 for model C, suggesting the fit of the models to data is similar. The marginal likelihoods are thus indecisive. We suggest that model C should be preferred, because of its biological plausibility.

### Analysis of the Budding Yeast Data Set

**FIg. 4.**
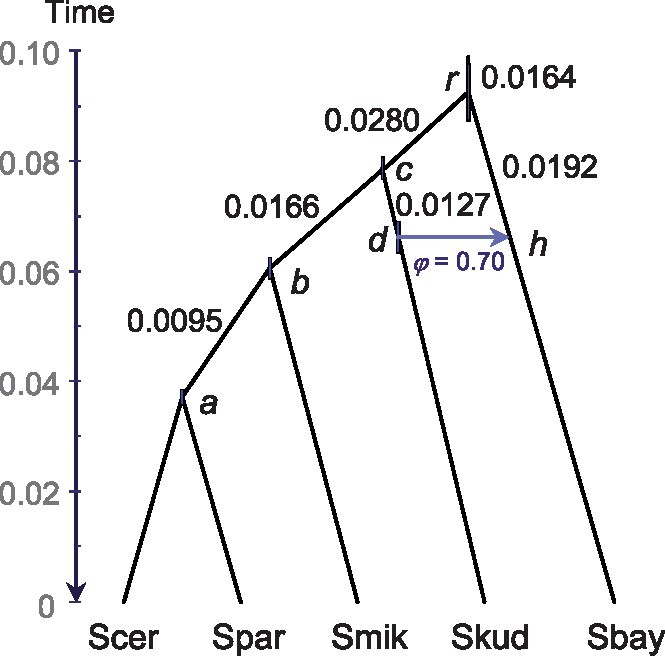
The species tree for five species of budding yeast, with one introgression event. The branches were drawn to reflect the posterior means of divergence/introgression times (*τ*s) from Bpp, with node bars showing the 95% HPD intervals, whereas posterior means of population sizes (*θ*s) are shown along the branches.

We fitted the MSci model of figure 4 to the data of 106 loci from 5 species of budding yeast. This model had a posterior probability of >95% in the PHyloNEt/mcmc-seq analysis of the same data by Wen and Nakhleh (2018). The φ estimate from Bpp was 0.70 (with the 95% HPD CI 0.56–0.83), compared with 0.75±0.06 in Wen and Nakhleh (2018). The small differences may be due to the use of different priors and the assumption of a constant *θ* across all populations in the PHyloNEt analysis. The results confirm the expectation that full-likelihood programs, if computationally feasible, should produce similar results. Running time for achieving an effective sample size (ESS) of 1,000 for φ was ∼3 min for Bpp using all 8 threads on a notebook, compared with ∼17 h for PHyloNEt using 32 threads on a computer server (Wen and Nakhleh 2018). If we make a 10-fold allowance for the fact that the model is fixed in Bpp while PHyloNEt spent computational efforts attempting changes to the model, this very roughly translates into a 100-fold difference in mixing/computational efficiency between the two programs (17×60/3×4/10=136).

### Variable Introgression across the Genome in the *Anopheles gambiae* Species Complex

**FIg. 5.**
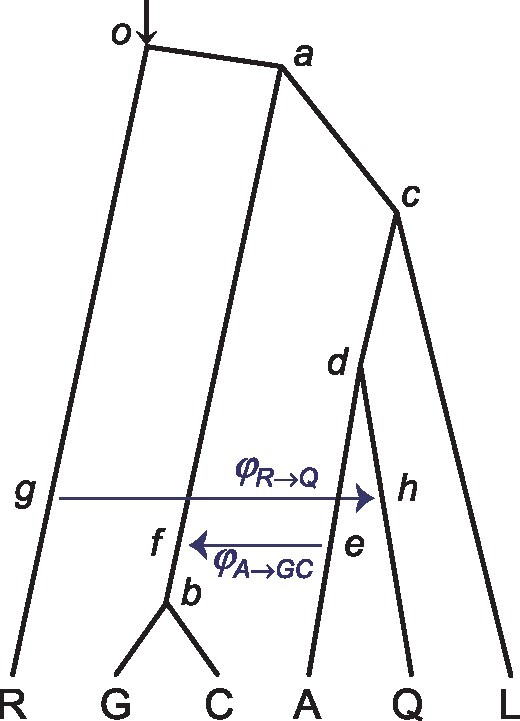
A species tree with two introgression events for the *Anopheles gambiae* species complex.

**FIg. 6.**
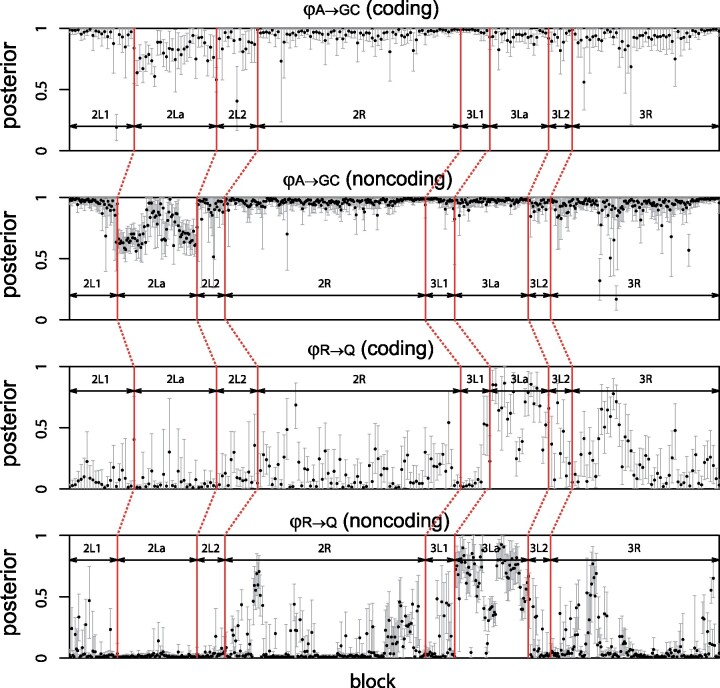
Posterior means and 95% HPD intervals of the introgression probabilities $\varphi_{A\to \mathrm{GC}}$ and $\varphi_{R\to Q}$ for the *Anopheles gambiae* species complex in Bpp analysis of the blocks. Each block consists of 100 loci, which are assumed to have the same φ at each hybridization node.

To examine the variation in introgression intensity across the *Anopheles* genome, we analyzed blocks of 100 loci, assuming the species tree of figure 5. Estimates of $\varphi_{A\to \mathrm{GC}}$ for the *A. arabiensis* → *A. gambiae* + *A. coluzzii* introgression and $\varphi_{R\to Q}$ for the *A. merus* → *A. quadriannulatus* introgression vary considerably across genomic regions or chromosomal arms (fig. 6). The probability $\varphi_{A\to \mathrm{GC}}$ is high (>0.5) in most blocks, whereas $\varphi_{R\to Q}$ is high in 3La and 3R.

We then merged the loci on the same chromosomal arms/regions to form 12 large coding and noncoding data sets, and analyzed them under the model of fig. 5 (table 1). We also sampled three sequences per locus to form data triplets for analysis using the maximum likelihood (ML) program 3S (Thawornwattana et al. 2018a, supplementary table S3, Supplementary Material online, GAR and RQO). For all autosomes, the introgression/migration rate from *A. arabiensis* to *A. gambiae* + *A. coluzzii* is very high (with $\varphi_{A\to \mathrm{GC}}>0.5$), whereas $M_{A\to G}$ ranges from 0.12 to 1.12. To reconcile the estimates from the two models, note that *M* is the expected number of migrants per generation, so that even a small *M* may mean a large number of migrants accumulated over many generations. As noted previously (Fontaine et al. 2015; Thawornwattana et al. 2018a), the autosomes are overwhelmed by the A→GC introgression so that all species tree methods that ignore gene flow infer incorrect species trees. In population genetic models of population subdivision, migration rates of M≪1 do not lead to substantial population subdivision. However, here *M* as low as 0.1 may have a significant impact on the species phylogeny if the species arose through radiative speciation events and the ancestral species had large sizes.

**Table 1.** Maximum Likelihood (3S) Estimates of Migration Rate (*M* = *Nm*) and Bayesian (Bpp) Estimates of Introgression Probability (φ) from the *Anopheles* Genomic Data.

Data Set	Loci	*A. arabiensis* → *A. gambiae + A. coluzzii*		*A. merus* → *A. quadriannulatus*	
		${\hat{M}}_{A\to G}$ (3S, GAR)	$\varphi_{A\to \mathrm{GC}}$ (Bpp)	${\hat{M}}_{R\to Q}$ (3S, RQO)	$\varphi_{R\to Q}$ (Bpp)
2L1 + 2 coding	3,585	0.319 0.372	0.943 (0.924, 0.963)	0.025 0.020	0.281 (0.242, 0.322)
2L1 + 2 noncoding	6,434	0.239 0.209	0.977 (0.967, 0.985)	0.000 0.000	0.002 (0.000, 0.003)
2La coding	2,776	0.915 1.120	0.731 (0.697, 0.766)	0.052 0.036	0.006 (0.002, 0.009)
2La noncoding	6,732	0.989 0.935	0.640 (0.627, 0.653)	0.000 0.000	0.001 (0.000, 0.002)
2R coding	6,849	0.511 0.477	0.966 (0.958, 0.975)	0.030 0.024	0.340 (0.295, 0.391)
2R noncoding	17,027	0.357 0.297	0.971 (0.961, 0.986)	0.007 0.007	0.222 (0.122, 0.342)
3L1 + 2 coding	1,747	0.168 0.340	0.939 (0.923, 0.956)	0.000 0.024	0.321 (0.271, 0.369)
3L1 + 2 noncoding	4,319	0.267 0.267	0.959 (0.951, 0.968)	0.000 0.003	0.330 (0.304, 0.356)
3La coding	1,998	0.866 0.790	0.931 (0.914, 0.947)	0.127 0.105	0.650 (0.619, 0.680)
3La noncoding	6,208	0.692 0.671	0.977 (0.970, 0.984)	0.021 0.020	0.544 (0.456, 0.700)
3R coding	4,977	0.393 0.365	0.945 (0.932, 0.958)	0.042 0.042	0.430 (0.371, 0.488)
3R noncoding	14,323	0.334 0.281	0.977 (0.971, 0.984)	0.009 0.009	0.030 (0.012, 0.062)

NOte.—ML estimates from 3S were obtained from two random samples of the GAR and RQO triplets, whereas the Bpp estimates (posterior means and 95% HPD intervals) used all 12 sequences at each locus.

Parameter $\varphi_{R\to Q}$ varied across chromosomal regions and was high for the inversion region 3La. Coding and noncoding loci produced highly consistent estimates of species trees, species divergence times, and population sizes (supplementary table S2, Supplementary Material online) (see also Thawornwattana et al. 2018a), but estimates of migration rate/introgression probability differed between the two data sets. The higher introgression rates for coding than noncoding loci in regions 3La, 2L1 + 2, and 3R suggest the intriguing possibility that the introgressed genes may have brought adaptive advantages, so that introgression is aided by natural selection. The functions of coding genes or exons that are most likely transferred across the species barriers could be examined to explore this hypothesis. There is overall consistency between estimates from the IM model in 3S and the MSci model in Bpp in that regions with high φ tend to have high *M* as well. Note that both φ and *M* reflect long-term effective gene flow, after the filtering of introgressed alleles by natural selection.

## Discussion

### Identifiability of MSci Models

If the probability distributions of the data are identical for two sets of parameter values (Θ and Θ′), with f(X|Θ)=f(X|Θ′) for all possible data *X*, then Θ is unidentifiable given data *X*. Previous studies of identifiability have mostly focused on the use of gene-tree topologies as data (Zhu and Degnan 2017; Degnan 2018). Note that a model unidentifiable given gene-tree topologies alone may be identifiable given gene trees with branch lengths or coalescent times, and that a model unidentifiable when one sequence is sampled per species may be identifiable when multiple samples per species are available (Yu et al. 2012; Pardi and Scornavacca 2015; Zhu and Degnan 2017).

A comprehensive examination of the identifiability issue under MSci is beyond the scope of this article. Here, we consider a few simple cases. First, the population size parameter *θ* is unidentifiable if at most one sequence per locus is sampled from that species or its descendant species and *τ*s associated with a hybridization event may also be unidentifiable. Consider the model of figure 2 and suppose the data consist of one sequence from each species. Then *θ_A_*, *θ_B_*, and *θ_C_*, as well as $\theta_{H_{1l}},\;\theta_{H_{1r}},\;\theta_{H_{2l}}$, and $\theta_{H_{2r}}$, are unidentifiable. In addition, $\tau_{H_{1}}$ and $\tau_{H_{2}}$ are unidentifiable. Parameters *τ_U_*, *τ_V,_ τ_S_*, *τ_R_*, and $\varphi_{H_{1}}$ and $\varphi_{H_{2}}$ are identifiable, as are $\theta_{R},\theta_{S},\theta_{U}$, and *θ_V._* In this case, the gene tree at any locus depends on whether sequence *c* takes the left path at *H*_1_ and enters species *U* (which happens with probability $\varphi_{H_{1}}$), or it takes the right path and enters species *H*_2_ (which happens with probability $1-\varphi_{H_{1}}$), but not on the age of the *H*_1_ node. The same applies to the path taken by sequence *c* at *H*_2_.

The most interesting case for the MSci model implemented here is where multiple sequences are sampled from each species at each locus, with multiple sites per locus. We speculate that the MSci model is identifiable on such data of sequence alignments as long as it is identifiable when the data consist of gene trees with coalescent times: Θ is identifiable using multilocus data *X* if and only if f(G,t|Θ)≠f(G,t|Θ′) for some *G* and ***t***. Note that identifiability implies statistical consistency for a full-likelihood method as implemented here. If the model is identifiable, the Bayesian parameter estimates will approach the true values when the number of loci approaches infinity.

Here, we note an interesting unidentifiability issue with model D of figure 1. Let Θ = ($\theta_{A},\theta_{B},\theta_{R},\theta_{X},\theta_{Y},\;\tau_{R},\tau_{X},\varphi_{X},\varphi_{Y}$) be the parameters of the model, and let Θ′ have the same parameter values as Θ except that $\theta \prime_{X}=\theta_{Y},\;\theta \prime_{Y}=\theta_{X}$, $\varphi \prime_{X}=1-\varphi_{X}$, and $\varphi \prime_{Y}=1-\varphi_{Y}$. Then f(G|Θ)=f(G|Θ′) for any Θ, *G*, and data configuration (with *n_A_* and *n_B_* sequences from *A* and *B*, respectively, say). Thus for every point Θ in the parameter space, there is a “mirror” point Θ′ with exactly the same likelihood. With Θ, a certain number of *A* sequences may take the left (upper) path at *X* (with probability $\varphi_{X}$) and enter population *XR*, coalescing at the rate $2/\theta_{X}$, whereas with Θ′, the same *A* sequences may take the right (horizontal) path (with probability $1-\varphi \prime_{X}=\varphi_{X}$) and enter population *YR*, coalescing at the rate $2/\theta \prime_{Y}=2/\theta_{X}$. The differences between the two scenarios are in the labeling only, with “left” and *X* under Θ corresponding to “right” and *Y* under Θ′, but the probabilities involved are exactly the same. The same argument applies to sequences from *B* going through node *Y*, and to sequences from *A* and *B* considered jointly. This is a case of the label-switching problem. Arguably Θ and Θ′ have the same biological interpretations concerning the relatedness of the sequences sampled from *A* and *B*. If the priors on $\varphi_{X}$ and $\varphi_{Y}$ are symmetrical, say beta(α,α), the posterior density will satisfy f(Θ|X)=f(Θ′|X) for all ***X***. Otherwise, the “twin towers” may not have exactly the same height.

Note that the label-switching kind of unidentifiability does not hinder the utility of the model. One can apply an identifiability constraint, such as φ<0.5, to remove the unidentifiability. However, in the general case of multiple bidirectional introgression events or multiple species on the species tree, it may be complicated to decide on the identifiability of the model.

Finally, we point out that there are many scenarios of data configurations and parameter settings in which some parameters are only weakly identifiable and very hard to estimate. For example, if *θ_C_* is very small relative to $\tau_{H_{1}}$ in figure 2, sequences from *C* will have coalesced before reaching node *H*_1_, so that only one *C* sequence passes *H*_1_ and the data will have little information about $\tau_{H_{1}},\theta_{H_{1l}},\theta_{H_{1r}},\tau_{H_{2}},\theta_{H_{2l}}$, and $\theta_{H_{2r}}$.

### Full-Likelihood and Summary Methods to Accommodate Introgression/Migration

Although biologically simplistic, the MSci and IM models offer powerful tools for analysis of genomic sequence data from closely related species, when cross-species gene flow appears to be the norm (Mallet et al. 2016; Martin and Jiggins 2017). Full-likelihood implementations of those models, including the ML (Zhu and Yang 2012; Dalquen et al. 2017) and Bayesian MCMC methods (Hey et al. 2018; Wen and Nakhleh 2018; Zhang et al. 2018), make efficient use of the information in the data and naturally accommodate phylogenetic uncertainties at individual loci caused by high sequence similarities (Edwards et al. 2016; Xu and Yang 2016). The complexity of those models means that large data sets with hundreds or thousands of loci may be necessary to obtain reliable parameter estimates, as indicated by our analyses of both simulated and real data. In this article, we have developed new MCMC proposal algorithms for MSci models (of types A–D of fig. 1) and have successfully applied them to analyze large data sets of over 10,000 loci (table 1). The algorithms appear to have good mixing efficiency. We suggest that this is a promising start, from which further improvements to the algorithms may be possible. Future work will include implementation of efficient MCMC proposals to move between MSci models, and a systematic examination of identifiability issues.

We note that the computational load for Bpp increases with an increase in the number of species, the number of hybridization events, the number of loci, the number of sequences per locus, or the number of sites per sequence. Increasing the number of species or hybridization events increases the number of parameters (as well as the number of models when the model is changing in the MCMC) so that the parameter space becomes much larger. Increasing the number of loci also increases the posterior search space since the MCMC has to sample in the space of gene trees for each locus (Flouri et al. 2018). In comparison, the number of sites per sequence has the least impact on the amount of computation. We found it helpful to make a distinction between computational efficiency of an MCMC algorithm, which reflects the computational time for each MCMC iteration, and mixing efficiency, which is measured by the ESS in parameter estimates for a given number of MCMC iterations. When the data set gets larger, particularly with more loci in the data set, the posterior for parameters becomes spiky, which in general leads to a deterioration of MCMC mixing efficiency, so that a greater number of MCMC iterations become necessary to produce estimates with acceptable precision. We conjecture that poor mixing is a more serious problem than poor computational efficiency for most MCMC algorithms in phylogenomics.

Our simulation suggests that under simple introgression scenarios, summary methods such as SNaQ and Hyde can produce as reliable estimates of the introgression probability as Bpp. However, full-likelihood methods provide measures of uncertainties and are applicable to complex introgression scenarios which are unidentifiable using summary methods. Summary methods are simple to implement, computationally efficient, and useful for analyzing large data sets. They can be used to generate hypotheses for further testing and estimation using Bpp. Furthermore, the current implementation of MSci in Bpp assumes the molecular clock and is unsuitable for distantly related species. Summary methods such as SNaQ use outgroups to root the tree without the need for the molecular clock.

### Variable Introgression Probability across the Genome

The models implemented here (fig. 1*A*–*C*) assume that the introgression probability or migration rate is constant among loci or across the genome. However, the impact of introgressed alleles on the fitness of the individual may strongly depend on the function of the genes in the introgressed region. Genes involved in cross-species incompatibilities are unlikely to be accepted in the recipient species. For example, crossing experiments between *A. arabiensis* and *A. gambiae* highlighted large differences between the chromosomes, with the X chromosome being most resistant to introgression, presumably because it harbors genes involved in cross-species sterility and inviability (Slotman et al. 2005). Differential selection across the genome means that the φ parameter should vary among loci. Note that φ in our models when estimated from genetic sequence data reflects the long-term combined effects of migration, recombination, and natural selection. It may be very different from the per-generation hybridization rate, which should apply to the whole genome.

In our analysis of the *Anopheles* genomic data, we used blocks of 100 loci to partially accommodate the variation in migration rate or introgression probability across chromosomal regions (fig. 6). We leave it to future work to implement MSci models with φ varying among loci. We note that many sequences per locus may be necessary to estimate locus-specific migration rates or introgression probabilities.

## Materials and Methods

### MCMC Proposals

We have adapted the five proposals in Rannala and Yang (2003) to accommodate hybridization nodes on the species tree and added another move to update the φ parameters.

Step 1. Change node ages on gene trees using a sliding window. Suppose the concerned gene-tree node is node *x* with age *t_x_* in population *X*, with parent node *p* in population *P* and two daughter nodes *u* and *v* in populations *U* and *V*, respectively. To propose a new node age $t_{x}^{*}$ first determine the bounds, $t_{L}<t_{x}^{*}<t_{U}$, with *t_U_* determined by the age of the parent node (*t_p_*) and *t_L_* by the age of the oldest daughter node: $t_{L}\geq \max(t_{u},t_{v})$. In addition, if the two daughter nodes are in different populations (with U≠V), *t_L_* must be older than the youngest common ancestor of populations *U* and *V* on the species tree.

Generate the new age $t_{x}^{*}$ by sampling around *t_x_*, reflected into the interval $\left(t_{L},t_{U}\right)$. The new node $x^{*}$ has to reside in a population that is descendant to the parent population *P* and ancestral to the child populations *U* and *V*. Among those target populations, we sample one uniformly. Given the sampled population for $x^{*}$, we sample the flags for the three branches: *p*–$x^{*},\;x^{*}$–*u*, and $x^{*}$–*v*. In each case, the two ends of the branch are already assigned a population. This move may cause large changes to the flags even though it does not change the gene-tree topology. For example, consider the change of *t_x_* in figure 2. Node *x* is in population *S*, with branch *x*–*v* having the flags l∅, since the branch passes *H*_1_ from the left and does not pass *H*_2_. Suppose the new age, generated in the interval (*t_u_*, *t_p_*), is $t_{x}^{*}>\tau_{R}$ so that the new node $x^{*}$ resides in *R*. The resampled flags for branch $x^{*}$–*v* may be *rr*, if the new branch passes both *H*_1_ and *H*_2_ from the right. The proposal ratio is given by the probabilities of sampling the flags at the *H* nodes.

Step 2. SPR move to change the gene-tree topology. This move cycles through the nonroot nodes on the gene trees. Suppose the node is *a*. We prune off its parent node *y*. The remaining part of the gene tree is called the backbone. We sample the new age ($t_{y}^{*}$) before reattaching the subtree *y*–*a* onto the backbone. This move always changes the node age *t_y_* but may not change the gene-tree topology.

First, we determine the bounds on the age of reattachment point: $t_{L}<t_{y}^{*}<t_{U}$. The maximum age is unbounded, whereas the minimum is $t_{L}\geq t_{a}$. However, if there are no branches on the backbone passing the population of node *a*, the reattachment point has to be in an ancestral species (in which there exists at least one branch on the backbone) and *t_L_* has to be greater. For example, clade *a* in figure 2 resides in population *B*, and if we prune off clade *a*, there will still be branches in *B* on the backbone for reattachment. In contrast, clade a′ resides in population $H_{1r}$, but if we prune off y′–a′, there will be no branches in population $H_{1r}$ for reattachment and the youngest ancestor of $H_{1r}$ with branches on the backbone is *V*, so that $t_{L}=\tau_{V}$.

We generate a new age $t_{y}^{*}$ around the current age (*t_y_*), reflected into the interval (*t_L_*, *t_U_*) if necessary, and then reattach *y* and clade *a* to a branch on the backbone at time $t_{y}^{*}$. A feasible target branch should cover $t_{y}^{*}$ and should at time $t_{y}^{*}$ be in a population ancestral to the population of *a*. We sample a target branch at random, either uniformly or with weights determined using local likelihoods.

In case the new branch $y^{*}$–*a* passes hybridization nodes, we sample the flags at each hybridization node, as in step 1. Suppose we prune off branch y′–a′ in figure 2 and the new age is $t_{y}^{\prime *}>\tau_{R}$. Then, we let branch $y^{\prime *}$–a′ go through $H_{2l}$ or $H_{2r}$ according to their probabilities. The proposal ratio is given by the number of target branches for reattachment and the probabilities for sampling the flags.

Step 3. Change *θ*s on the species tree using a sliding window. This step is the same as in Rannala and Yang (2003).

Step 4. Change *τ*s on the species tree using a variant of the rubber-band algorithm (Rannala and Yang 2003). We generate a new age ($\tau^{*}$) around the current age, reflected into the interval $\left(\tau_{L},\tau_{U}\right)$, determined using the ages of the parent nodes and daughter nodes on the species tree. Next we change the ages of the affected nodes on the gene trees using the rubber-band algorithm. An affected node has age in the interval $\left(\tau_{L},\tau_{U}\right)$ and resides in the current population (with age *τ*) or the two daughter populations (if a speciation node is changed), or in the two current populations (*H_l_* and *H_r_*) and the daughter population (if an *H* node is changed). For example, to change *τ_S_*, the bounds are $\left(\tau_{H_{2}},\tau_{R}\right)$, and the affected nodes on the gene tree of figure 2 are in species *S*, *U*, and *H*_2_. These are *x*, *u*, and *w*. To change $\tau_{H_{2}}$ the bounds are $\left(\tau_{H_{1}},\tau_{V}\right)$ and the affected nodes are in species $H_{2l},\;H_{2r}$, and $H_{1r}$, and are a′. The proposal for changing node ages on the gene tree given the bounds is as in (Rannala and Yang 2003, eqs. A7 and A8).

Step 5. Rescale all node ages on the species tree and on the gene trees using a mixing step (a multiplier) (Rannala and Yang 2003).

Step 6. Change the introgression probability φ for each introgression event using a sliding window. This step affects the gene-tree density, but not the sequence likelihood.

The sliding window used in Bpp is the Bactrian move with the triangle kernel (Yang and Rodriguez 2013; Thawornwattana et al. 2018b). Step lengths are adjusted automatically during the burn-in, to achieve an acceptance rate of ∼30% (Yang and Rodriguez 2013).

### Simulation Study

We conducted three sets of simulations. The first set includes multiple sequences from each species and examines Bpp estimation of parameters in the MSci model and the impact of the number of loci, the introgression probability φ, and the species tree model. The second set compares Bpp with two summary methods: SNaQ (Solis-Lemus and Ane 2016; Solis-Lemus et al. 2017) and Hyde (Blischak et al. 2018), using one sequence per species. The third set explores the performance of Bpp when the model is unidentifiable using SNaQ and Hyde.

For the first set of simulations, multilocus data sets were simulated under the MSci models A and C of figure 1 and then analyzed using Bpp to examine the precision and accuracy of parameter estimation. For model A, we used $\tau_{R}=0.03$, $\tau_{S}=0.02,\;\tau_{T}=0.02$, and $\tau_{H}=0.01$. For model C, we used $\tau_{R}=0.03$ and $\tau_{S}=\tau_{T}=\tau_{H}=0.01$. We used two values of φ (0.1 and 0.5) and two values of *θ* (0.001 and 0.01), applied to all populations. Each data set consisted of 10, 100, or 1,000 loci, and at each locus, 10 sequences were sampled from each species (with 30 sequences in total). The sequence length was 500 sites.

Data were generated using the “simulate” option of Bpp. Gene trees with branch lengths (coalescent times) were simulated under the MSci model. Then, sequences were “evolved” along the branches of the gene tree according to either the JC (Jukes and Cantor 1969) or the GTR + Γ (Yang 1994a, 1994b) models, and the sequences at the tips of the gene tree constituted the data at the locus. In the GTR + Γ model, the GTR parameters varied among loci according to estimates obtained for chromosomal arm 2L from the *A.* *gambiae* species complex (Thawornwattana et al. 2018a). The base-frequency parameters were generated from a Dirichlet distribution $\left(\pi_{T},\pi_{C},\pi_{A},\pi_{G})∼\text{Dir}(25.18,20.50,25.22,20.38\right)$. The GTR exchangeability parameters (Yang 1994a) were (a,b,c,d,e,f)∼Dir(7.59,3.23,2.95,2.93,2.93,7.57). The overall rates for loci varied according to a gamma distribution *G*(5, 5), whereas the rates for sites at the same locus varied according to the gamma distribution with mean one, G(α,α) (Yang 1994b), with the shape parameter *α* sampled from *G*(20, 4).

The number of replicates was 10. Thus, with two trees (*A* and *C*), two φ values (0.1 and 0.5), two *θ* values (0.001 and 0.01), two mutation models (JC and GTR + Γ), and three data sizes (10, 100, and 1,000 loci), a total of 480=2×2×2×2×3×10 replicate data sets were generated.

Each data set was analyzed using Bpp. The JC model was always assumed whether the data were simulated under JC or GTR + Γ. Inverse-gamma priors were assigned on parameters *θ* and *τ*_0_ (the root age), with the shape parameter 3 and the prior mean equal to the true value: IG(3, 0.02) for θ=0.01 and IG(3, 0.002) for θ=0.001, and $\tau_{0}∼$ IG(3, 0.06). The inverse-gamma distribution with shape parameter *α *= 3 has the coefficient of variation 1 and constitutes a diffuse prior. The uniform prior U(0,1) was used for φ.

Pilot runs were used to determine the suitable chain length, and then the same settings (such as the burn-in, the number of MCMC iterations, and the sampling frequency) were used to analyze all replicates. Convergence was assessed by running the same analysis multiple times and confirming consistency between runs (Yang 2015; Flouri et al. 2018).

The second set of simulation was to compare Bpp with summary methods. Most methods are designed to test for the presence of gene flow (hybridization or migration) (Degnan 2018). Here, we used two methods that can estimate the introgression probability under a fixed introgression model: SNaQ (Solis-Lemus and Ane 2016) implemented in the program PhyloNetworks (Solis-Lemus et al. 2017) and Hyde (Blischak et al. 2018). The basic algorithms for SNaQ and Hyde are formulated for the case of three species with one or two outgroup species used to root the tree. SNaQ uses the proportions of the three gene-tree topologies, based on the observation that the probabilities for the two mismatching gene trees (which have different topologies from the species tree) are the same if there is deep coalescent but no gene flow while they are different if there is gene flow as well (Yu et al. 2014). Hyde uses the proportions of the three parsimony-informative site patterns pooled across loci or genomic regions (*xxyy*, *xyxy*, and *xyyx*), based on the observation that the probabilities for the two “mismatching” site patterns (*xyxy* and *xyyx*) are the same if there is deep coalescent but no gene flow while these are different if there is gene flow as well (Green et al. 2010).

We used model A of figure 1, plus two outgroup species *D* and *E*, to simulate *L *=* *10, 100, or 1,000 loci, with one sequence per species per locus. The data were then analyzed using SNaQ and Hyde, as well as Bpp. The JC model was used both to simulate and to analyze the data. For SNaQ, gene trees were inferred using RAxML (Stamatakis et al. 2012). For Bpp, the point estimates (posterior means) of φ were used for comparison even though estimates for all parameters, with CIs, were produced.

The third set of simulation explores the performance of Bpp under models that are unidentifiable using SNaQ and Hyde. An MSci model may be identifiable given the gene trees with coalescent times but unidentifiable given gene-tree topologies only (Degnan 2018). We simulated and analyzed data using Bpp under two models: model D of figure 1 with bidirectional introgression between species *A* and *B* and the model of figure 2 (referred to as model 2H), with three species and two introgression events. Under model D, there is only one gene tree between two species so that its frequency is uninformative and SNaQ is not applicable, and nor is Hyde. Under model 2H, frequencies of three gene trees or three site patterns cannot be used to estimate two introgression probabilities and two internal branch lengths: It is thus impossible to apply SNaQ and Hyde to such data.

For model D, we used the following parameter values: $\tau_{R}=0.01,\tau_{X}=\tau_{Y}=0.005,\varphi_{X}=0.1,\varphi_{Y}=0.3$, and θ=0.01 for all populations. We simulated 10 replicate data sets, each of 10, 100, or 1,000 loci. At each locus, we sampled 10 sequences per species (20 sequences in total), with the sequence length to be 500. The JC mutation model was used both to simulate and to analyze data by Bpp. Note that there is an interesting identifiability issue (or label-switching issue) with model D, such that the two sets of parameters $\Theta =(\theta_{A},\theta_{B},\theta_{R},\theta_{X},\theta_{Y},\tau_{R},\tau_{X},\varphi_{X},\varphi_{Y})$ and $\Theta \prime =(\theta_{A},\theta_{B},\theta_{R},\theta_{Y},\theta_{X},\tau_{R},\tau_{X},1-\varphi_{X},1-\varphi_{Y})$ are unidentifiable (see Discussion). Thus, an identifiability constraint should be applied, such as $\varphi_{X}<0.5$. We ran the MCMC without any constraint, but the MCMC sample was preprocessed, with Θ replaced by Θ′ if the sampled value for $\varphi_{X}>0.5$, before the posterior summary was generated.

For model 2H (fig. 2), the following parameter values were used: $\tau_{R}=0.04,\tau_{S}=0.03,\tau_{U}=0.02,\tau_{V}=0.03,\tau_{H_{1}}=0.01,\tau_{H_{2}}=0.02,\varphi_{H_{1}}=0.1,\varphi_{H_{2}}=0.5$, and θ=0.01 for all populations. As above, 10 sequences per species were generated, with 30 sequences per locus. The sequence length was 500. The JC model was used both to simulate and to analyze the data.

### Analysis of the Purple Cone Spruce Data Sets

We reanalyzed sequence data concerning the origin of the purple cone spruce *Picea purpurea* (P) from the Qinghai–Tibet Plateau, hypothesized to have originated through homoploid hybridization between *P. wilsonii* (W) and *P. likiangensis* (L) (Sun et al. 2014). The data were generated by Sun et al. (2014). To make the computation feasible for the *beast program, Zhang et al. (2018) constructed and analyzed two nonoverlapping data subsets (data sets 1 and 2), each with 40, 30, and 30 phased sequences for P, W, and L, respectively, at 11 autosomal loci. We analyzed these data sets for comparison with the analysis of Zhang et al. (2018) using *beast. We also used Bpp to analyze the “Full” data set from which data sets 1 and 2 were sampled, with 112, 100, and 120 sequences per locus for the same 11 loci.

The species tree of figure 3 was assumed (Sun et al. 2014). The priors were $\tau_{0}∼$ IG(3,0.004), θ∼IG(3,0.003), and φ∼U(0,1). Rates for loci were either constant or had a Dirichlet distribution with *α *= 2 (Burgess and Yang 2008). We used a burn-in of 32,000 iterations and took 10^5^ samples, sampling every 10 iterations. The program was run at least twice for each analysis, to check for consistency between runs. Each run (on a single core) took ∼5 days.

Marginal likelihood for models A–C (fig. 1) was calculated using thermodynamic integration with Gaussian quadrature (Lartillot and Philippe 2006; Rannala and Yang 2017), with 16 quadrature points.

### Analysis of the Budding Yeast Data Set

We analyzed a budding yeast data set with 106 loci and five species: *Saccharomyces cerevisiae* (Scer), *Saccharomyces paradoxus* (Spar), *Saccharomyces mikatae* (Smik), *Saccharomyces kudriavzevii* (Skud), and *Saccharomyces bayanus* (Sbay). This is a subset of the data set published by Rokas et al. (2003) and previously analyzed by Wen and Nakhleh (2018). The species tree or MSci model is shown in figure 4, with a Skud → Sbay introgression. We used inverse-gamma priors IG(3, 0.04) for *θ*s and IG(3, 0.2) for *τ*_0_, and φ∼U(0,1).

### Analysis of the Genomic Data from the *A. gambiae* Species Complex

We used the coding and noncoding loci compiled by Thawornwattana et al. (2018a) from the genomic sequences for six species in the *A. gambiae* species complex: *A. gambiae* (G), *A. coluzzii* (C), *A. arabiensis* (A), *A. melas* (L), *A. merus* (R), and *A. quadriannulatus* (Q) (Fontaine et al. 2015). There are 12 sequences per locus, with two sequences per species.

We analyzed blocks of 100 loci, as in Thawornwattana et al. (2018a), and then combined loci for each of the eight chromosomal arms/regions: 2L1, 2La (the inversion region on 2L), 2L2, 2R, 3L1, 3La (the inversion region on 3L), 3L2, and 3R. Since our objective was to estimate the introgression probability for the autosomes, the X chromosome was not used. The species tree is in figure 5, from Thawornwattana et al. (2018a, figure 6). The priors were $\tau_{0}∼\text{IG}(3,0.2)$ with mean 0.1 for the age of the root, θ∼IG(3,0.04) with mean 0.02, and φ∼U(0,1). We used a burn-in of 16,000 iterations, and took $5\times 10^{5}$ samples, sampling every 2 iterations. Pilot runs suggest that this generates ESS > 1,000. Each analysis of the block took a few hours, whereas the analysis of the 12 large combined data sets of table 1 each took 1–2 weeks.

For comparison, we used the ML program 3S (Zhu and Yang 2012; Dalquen et al. 2017) to estimate the migration rate *M* = *Nm* under the IM model. The implementation assumes three species (1, 2, and 3, say), with three sequences per locus. We sampled three sequences, with half of the loci having the “123” configuration, a quarter with “113,” and another quarter with “223” (Thawornwattana et al. 2018a). We generated two replicate data sets by sampling the GAR and RQO triplets to estimate the migration rate $M_{A\to G}$ and $M_{R\to Q}$ (fig. 5). Although limited to three sequences, 3S can use tens of thousands of loci and each run took a few minutes.

## Software Availability

The MCMC algorithms described in the article are implemented in Bpp Version 4 (Yang 2015; Flouri et al. 2018), available at https://github.com/bpp. The python3 code and scripts for simulating and analyzing the sequence data and for making plots using ggplot are available at https://github.com/brannala/NetworkMSCSimulations.

## Supplementary Material

msz296-Supplementary_Data

## Appendix: Extended Newick Notation for the MSci Model

We use the extended Newick notation (Cardona et al. 2008) to represent the MSci model in the Bpp program. The parenthesis notation “(A,B)S” specifies two branches from the speciation node *S* to two daughter species *A* and *B*, whereas “(A)H” specifies one branch from *H* to *A*. Every branch is represented once. Each tip species occurs once. Internal nodes for speciation nodes may and may not be labeled, but hybridization (*H*) nodes must be labeled. In models A–C of figure 1, each *H* node occurs twice in the notation, once as a label for an ancestral node and another time as a tip, and the introgression probability φ is identified with the ancestral node (whereas its “mirror” tip node has 1−φ). Thus, models A–C of figure 1 are represented as ((A,(C)H)S,(H,B)T)R, with parameter φ assigned to the *SH* branch and 1−φ to the *TH* branch. The extended Newick notation is not unique. The representation ((A,H)S,((C)H,B)T)R specifies an equivalent model, with parameter φ assigned to branch *TH* and 1−φ to branch *SH*.

The three types of models in figure 1 (A–C) are distinguished using the metadata variable “tau-parent,” which is assigned the value “yes” or “no” depending on whether the parent node has an age (*τ*) distinct from that of the hybridization node. Thus, models A–C of figure 1 are represented as

(A):((A,(C)H[&tau‐parent=yes])S,(H[&tau‐parent=yes],B)T)R.

(B):((A,(C)H[&tau‐parent=no])S,(H[&tau‐parent=yes],B)T)R.

(C):((A,(C)H[&tau‐parent=no])S,(H[&tau‐parent=no],B)T)R.

Model D (bidirectional introgression) differs from models A–C in that each of nodes *X* and *Y* has two parent nodes and two daughter nodes. The model is represented as ((A,(B)Y)X,(X)Y)R. The notation is again not unique, and equivalent representations include (((A)X,B)Y,(Y)X)R or more concisely, ((X,B)Y,(A,Y)X)R. In both notations, the φ parameter is assigned to the branch with an older parent, whereas the horizontal branch has 1−φ.

As a more complex example, the species graph for the *Anopheles* mosquitoes of figure 5 is represented as

((R,(Q)h[&tau‐parent=no])g,(f[&tau‐parent=yes],(((((G,C)b)f[&tau‐parent=no],A)e,h[&tau‐parent=yes])d,L)c)a)o.
