# Correction to: A Bayesian Implementation of the Multispecies Coalescent Model with Introgression for Phylogenomic Analysis

**DOI:** 10.1093/molbev/msac245

**Published:** 2022-11-15

This is a correction to: Tomáš Flouri, Xiyun Jiao, Bruce Rannala, Ziheng Yang, A Bayesian Implementation of the Multispecies Coalescent Model with Introgression for Phylogenomic Analysis, Molecular Biology and Evolution, Volume 37, Issue 4, April 2020, Pages 1211–1223, https://doi.org/10.1093/molbev/msz296

In the originally published version of this manuscript, the supplementary data posted is incorrect and does not include Tables S1 and S2. The correct supplementary pdf file including figures S1-S10 and tables S1 and S2 is given here.

These details have been corrected only in this correction notice to preserve the published version of record.

[2020FlourisMSci-SI.pdf]

