# Supplementary material for: A Bayesian Implementation of the Multispecies Coalescent Model with Introgression for Phylogenomic Analysis
Source: Mol Biol Evol. 2019 Dec 6;37(4):1211–23. doi: 10.1093/molbev/msz296 (PMC7086182; doi:10.1093/molbev/msz296)
Supplement: msz296-Supplementary_Data [file molbev_37_4_1211_s0.zip › 2020FlourisMSci-SI.pdf]

Supplementary material

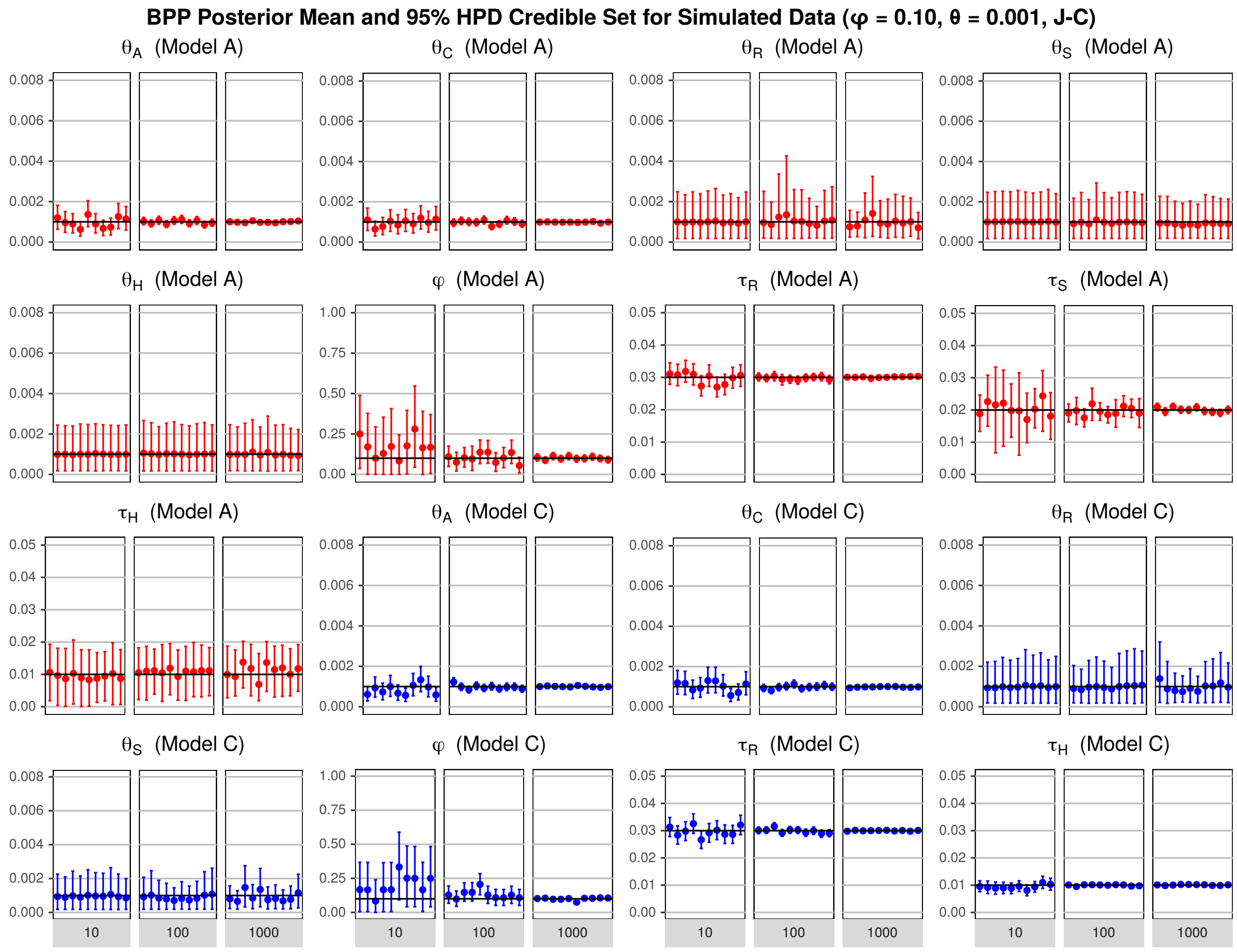

Figure S1: Posterior means and 95% HPD credibility intervals (CI) of parameters under models A and C of Fig. 1 in 10 replicate datasets, each of 10, 100, or 1000 loci, simulated under the parameter combinations: $\varphi = 0.1$, $\theta = 0.001$, and the JC mutation model. The horizontal line represents the true parameter value. Note that there are 13 parameters under model A ($\theta_A, \theta_B, \theta_C, \theta_R, \theta_S, \theta_T, \theta_{H_l}, \theta_{H_r}, \tau_R, \tau_S, \tau_T, \tau_H, \varphi$), and 9 under model C ($\theta_A, \theta_B, \theta_C, \theta_R, \theta_S, \theta_T, \tau_R, \tau_S, \varphi$). Due to the symmetry of the experimental design, the results for some parameters are identical so that only the non-redundant results are shown to save space: for example, θ_A is shown but θ_B is not.

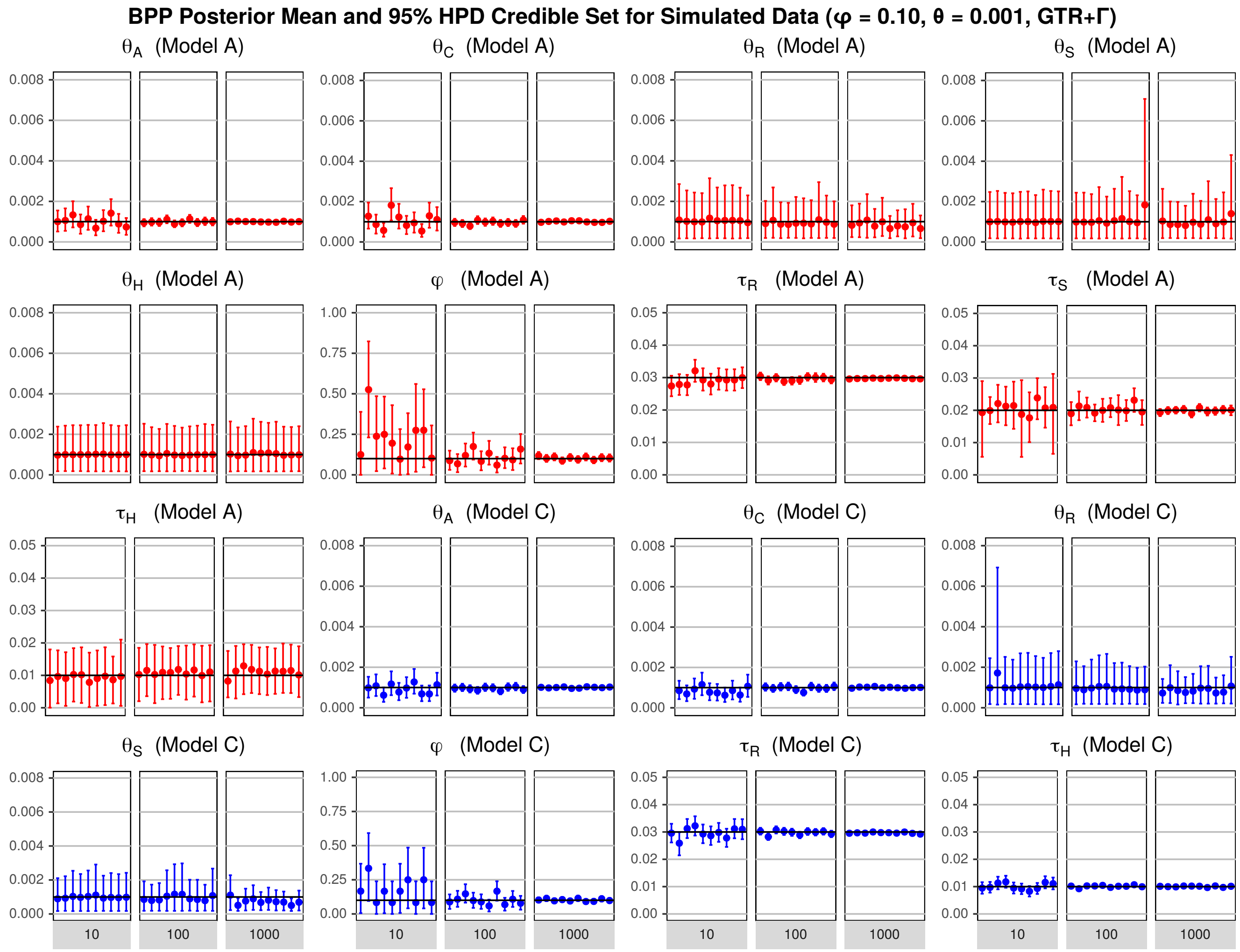

Figure S2: Simulation results for the combination $\varphi = 0.1$, $\theta = 0.001$, and the GTR+Γ mutation model. See legend to Fig. S1.

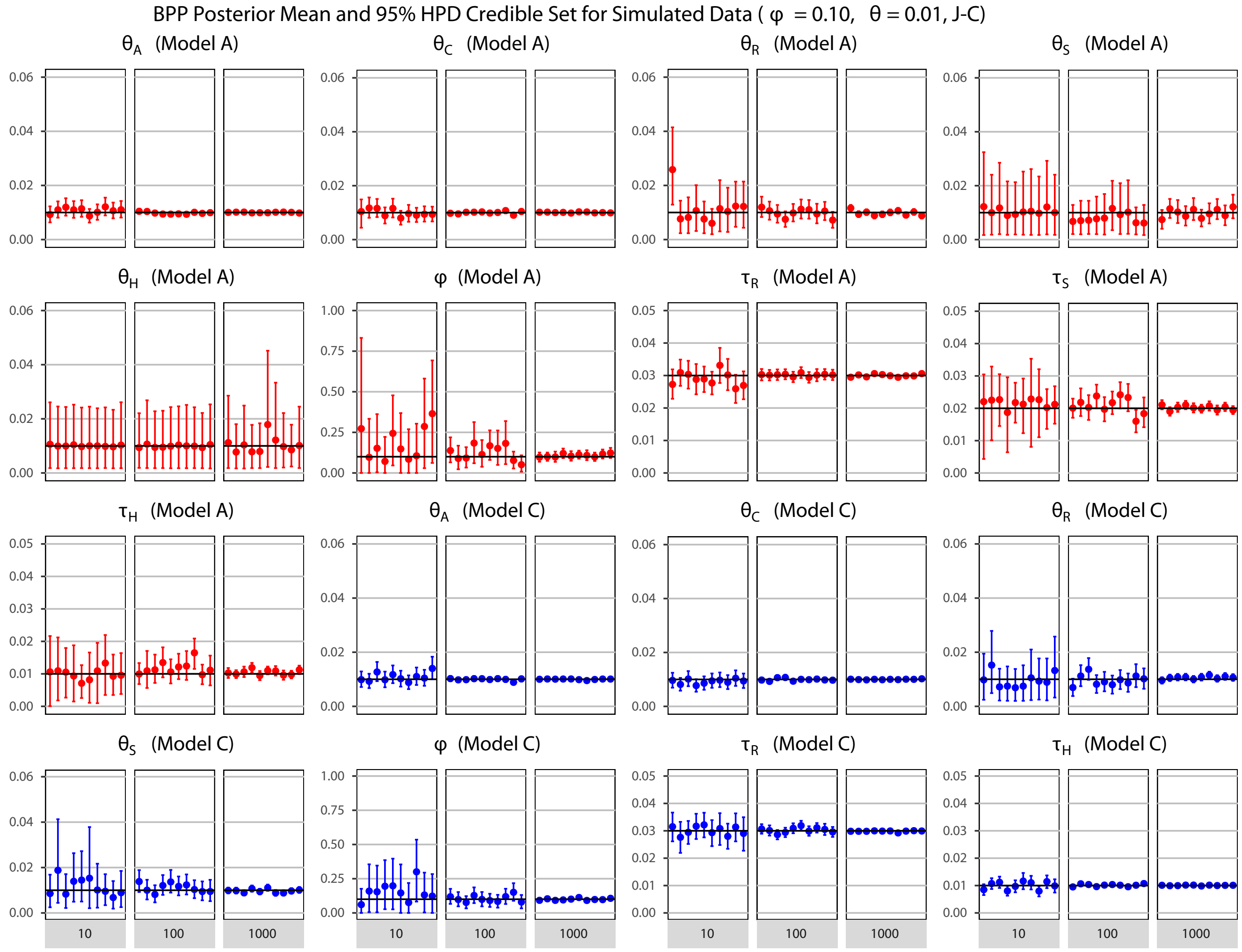

Figure S3: Simulation results for the combination $\varphi = 0.1$, $\theta = 0.01$, and the JC mutation model. See legend to Fig. S1.

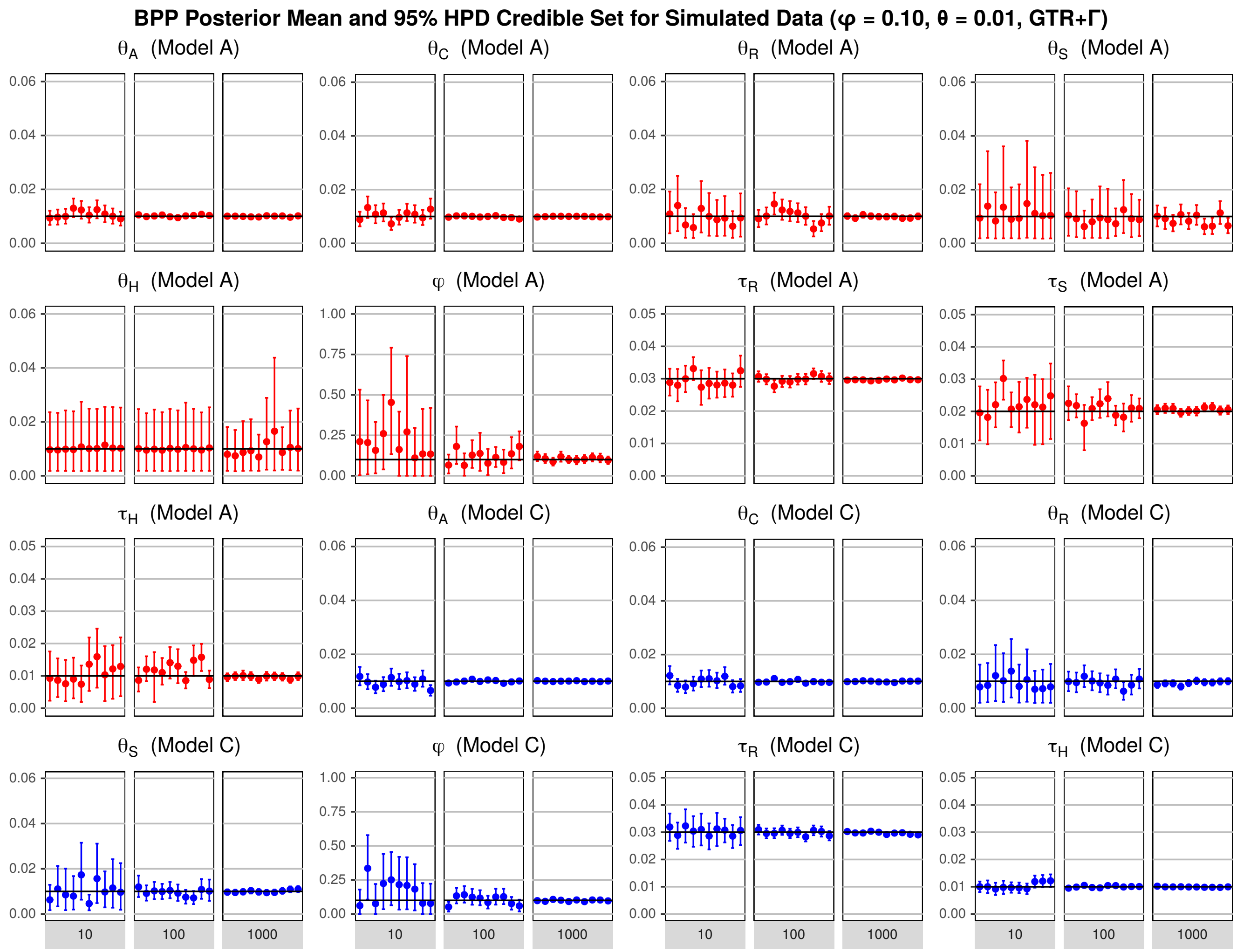

Figure S4: Simulation results for the combination $\varphi = 0.1$, $\theta = 0.01$, and the GTR+Γ mutation model. See legend to Fig. S1.

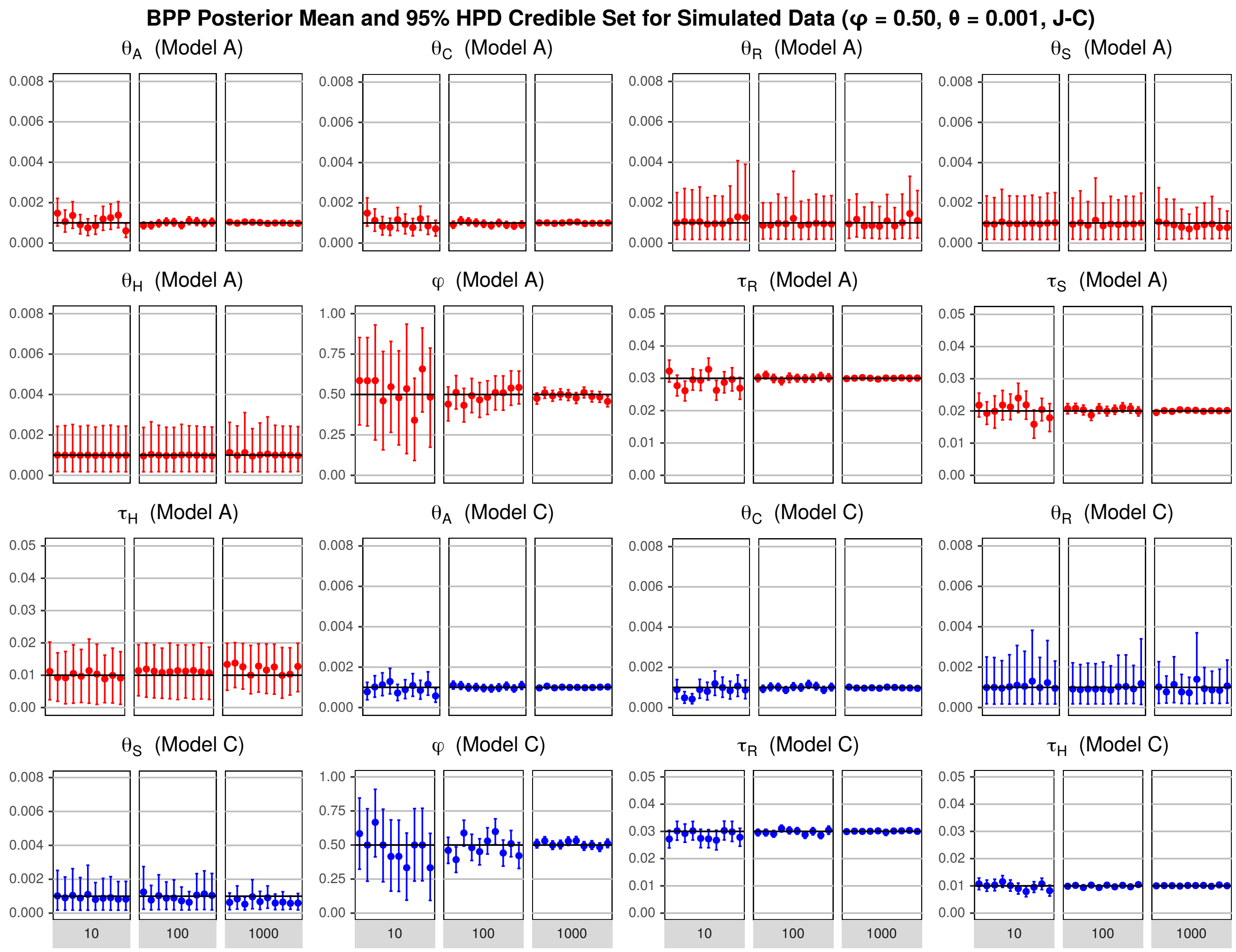

Figure S5: Simulation results for the combination $\varphi = 0.5$, $\theta = 0.001$, and the JC mutation model. See legend to Fig. S1.

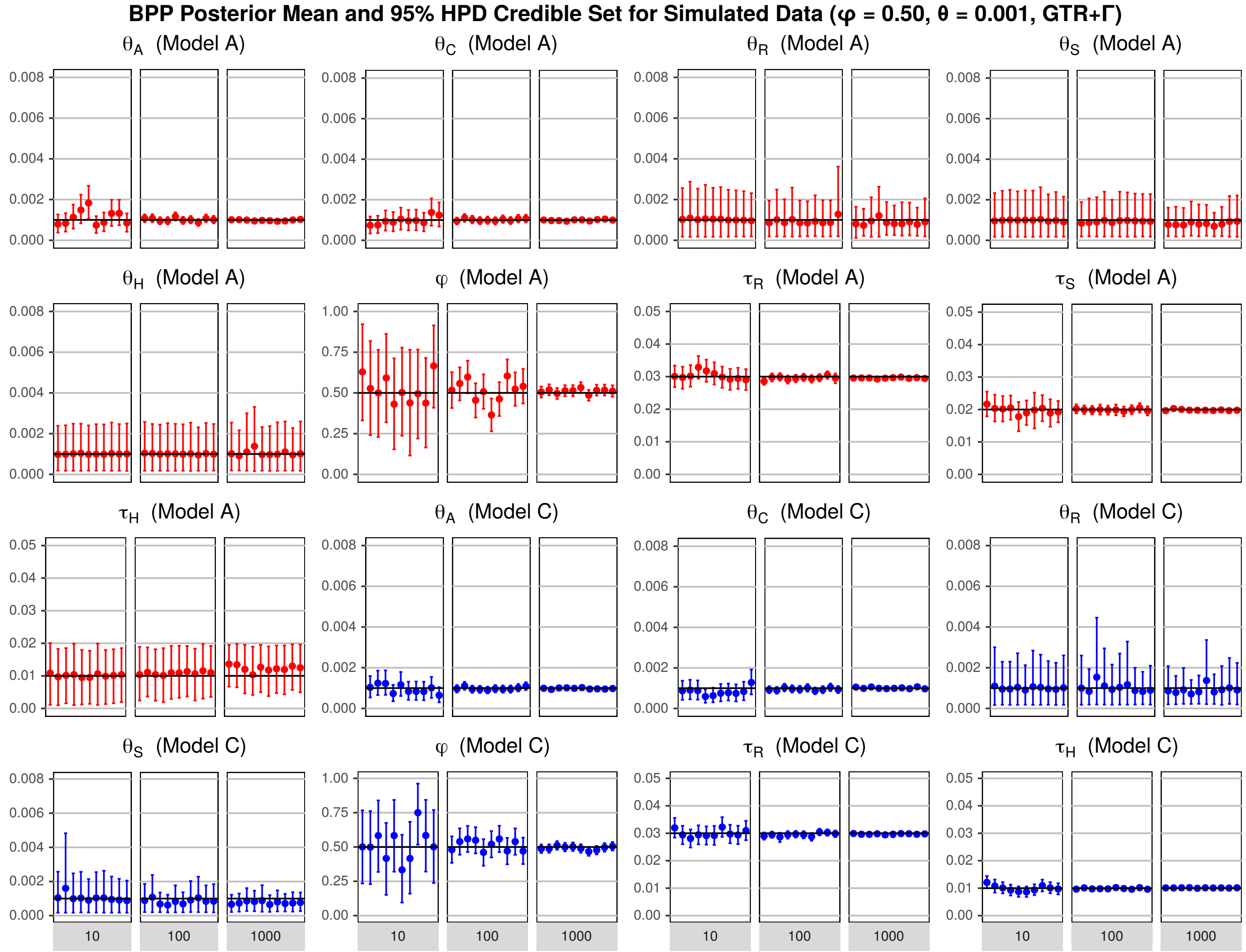

Figure S6: Simulation results for the combination $\varphi = 0.5$, $\theta = 0.001$, and the GTR+Γ mutation model. See legend to Fig. S1.

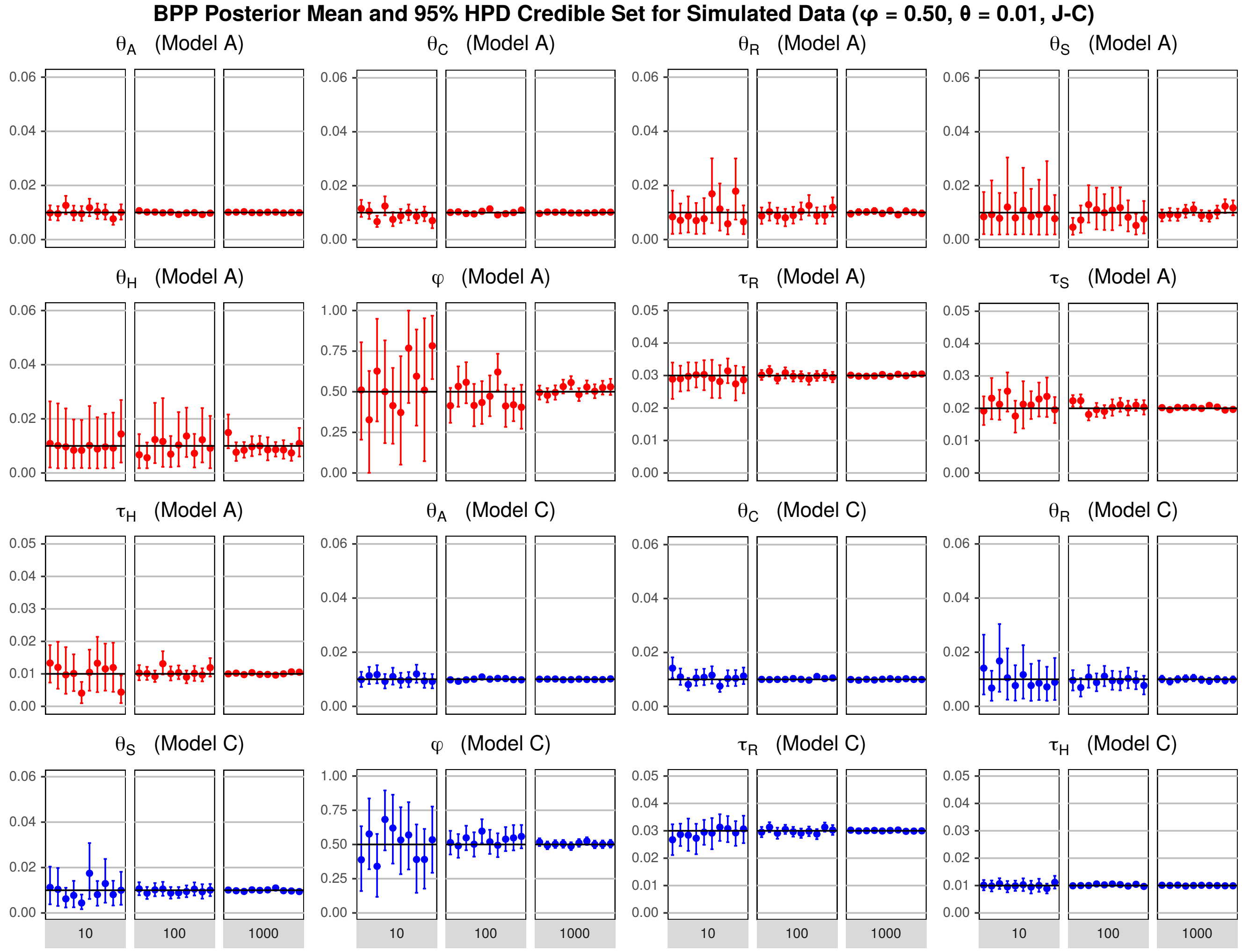

Figure S7: Simulation results for the combination $\varphi = 0.5$, $\theta = 0.01$, and the JC mutation model. See legend to Fig. S1.

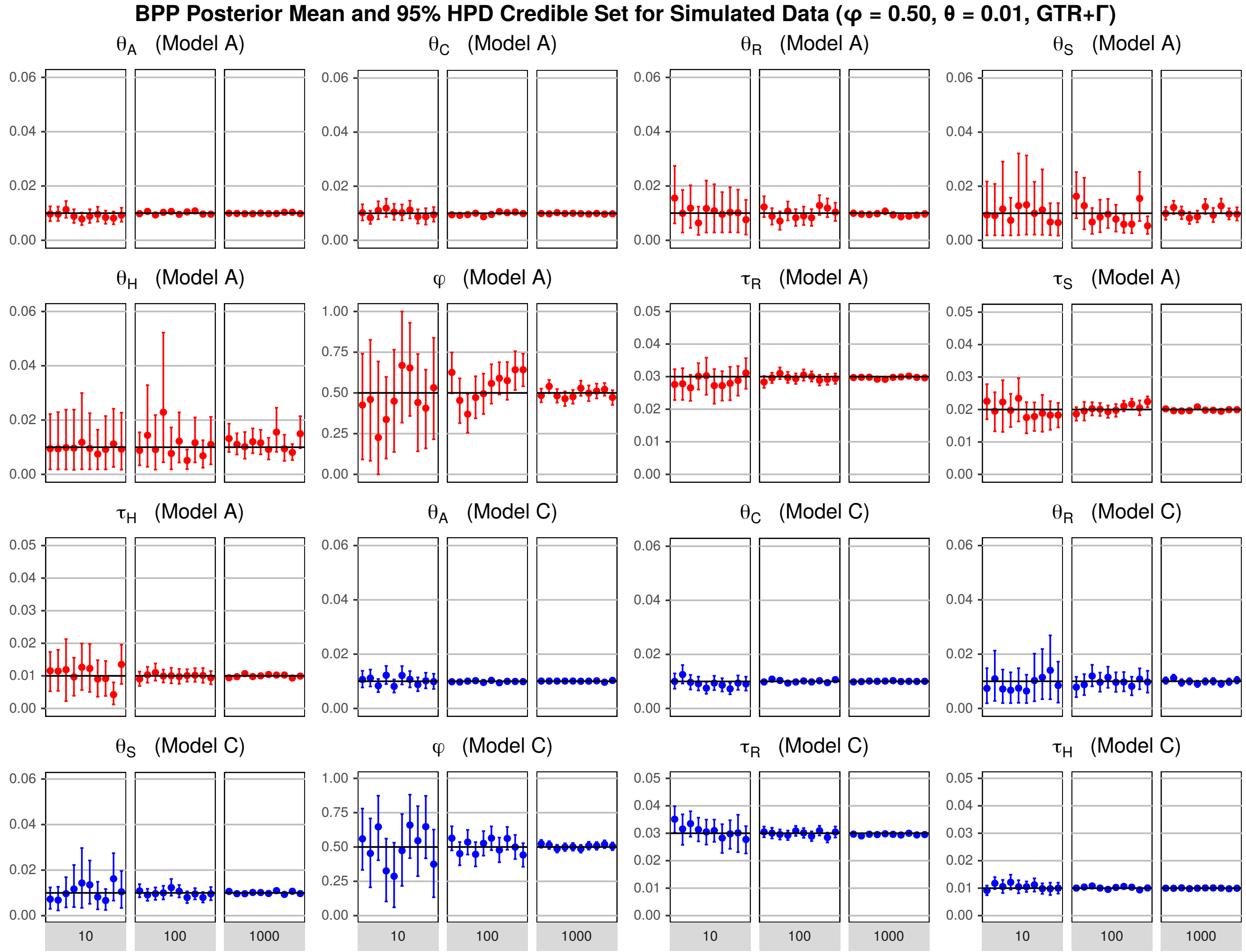

Figure S8: Simulation results for the combination $\varphi = 0.5$, $\theta = 0.01$, and the GTR+Γ mutation model. See legend to Fig. S1.

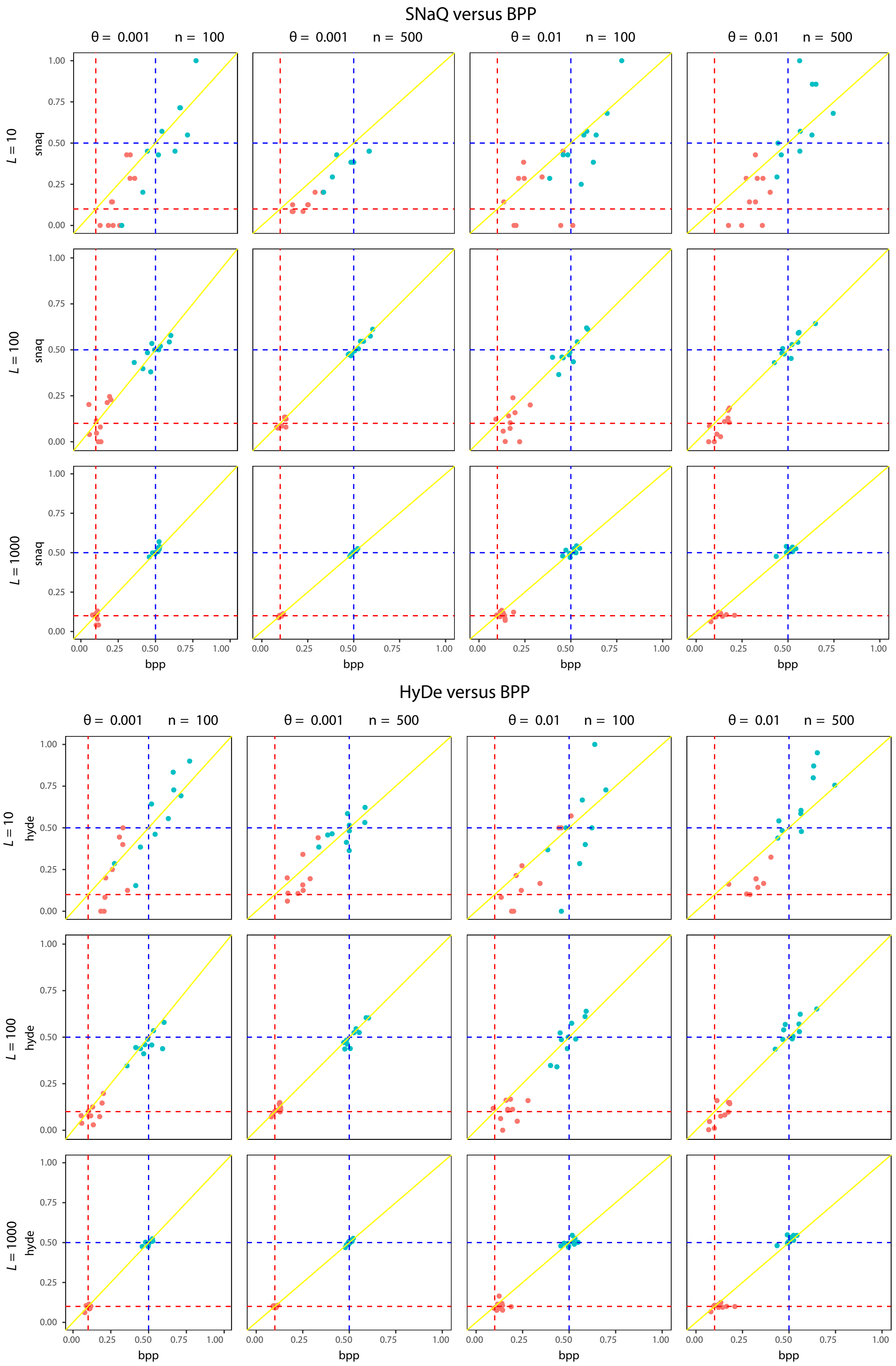

Figure S9: Estimates of φ using SNaQ and HyDe plotted against those from BPP on simulated datasets. Model A for three species (Fig. 1A) was used to simulate 10 replicate datasets, using $\varphi = 0.1$ or 0.5, $\theta = 0.001$ or 0.01, the number of loci $L = 10, 100$, or 1000, and sequence length $n = 100$ or 500. The dotted lines indicate the true values.

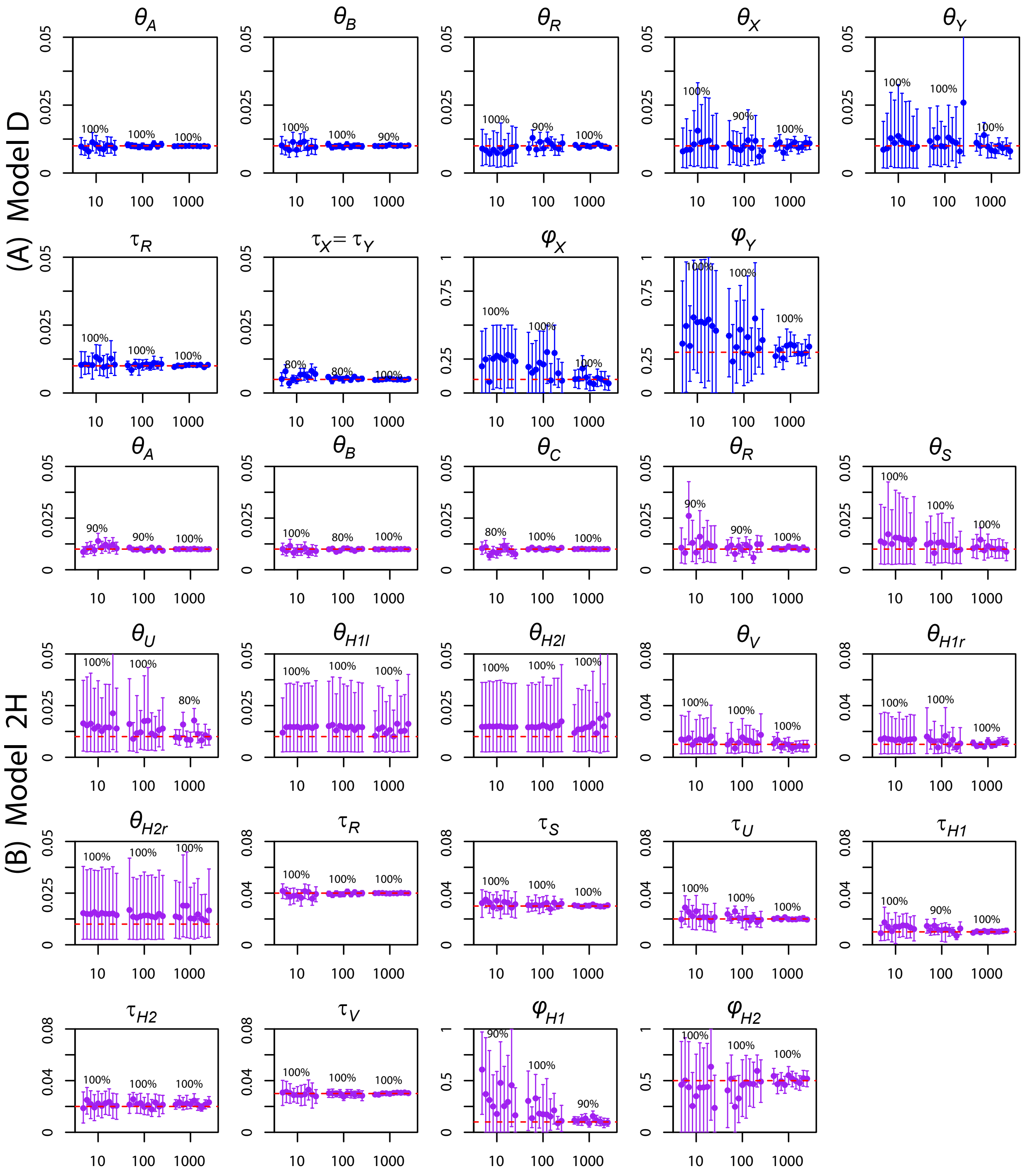

Figure S10: Posterior means and 95% HPD CIs of parameters under model D of Fig. 1 (blue) and model 2H of Fig. 2 (purple) in 10 replicate datasets, each of 10, 100, or 1000 loci. The numbers above the CI bars are the coverage or the proportion of replicate datasets in which the CI bar includes the truth. The horizontal lines represent the true parameter values.

Table S1. Estimates of parameters under the MSci models A and C of Fig. 1 (posterior means and 95% HPD intervals) for the purple cone spruce datasets

	Dataset1, model A, one rate	Dataset1, model A, Dir (2)	Dataset2, model A, one rate	Dataset2, model A, Dir (2)
φ	0.34103 (0.13314, 0.58684)	0.32123 (0.12233, 0.57622)	0.43591 (0.15909, 0.69538)	0.40490 (0.13650, 0.66916)
τ_R	0.00142 (0.00105, 0.00180)	0.00133 (0.00098, 0.00169)	0.00129 (0.00098, 0.00161)	0.00119 (0.00090, 0.00149)
τ_D	0.00011 (0.00001, 0.00024)	0.00009 (0.00001, 0.00022)	0.00005 (0.00001, 0.00011)	0.00005 (0.00001, 0.00010)
τ_E	0.00025 (0.00005, 0.00049)	0.00026 (0.00005, 0.00050)	0.00022 (0.00002, 0.00044)	0.00024 (0.00003, 0.00045)
τ_H	0.00007 (0.00000, 0.00020)	0.00006 (0.00000, 0.00019)	0.00002 (0.00000, 0.00008)	0.00002 (0.00000, 0.00008)
θ_W	0.00153 (0.00037, 0.00302)	0.00146 (0.00035, 0.00294)	0.00083 (0.00027, 0.00160)	0.00084 (0.00027, 0.00162)
θ_P	0.01197 (0.00030, 0.03277)	0.01083 (0.00030, 0.03137)	0.00599 (0.00031, 0.01822)	0.00593 (0.00029, 0.01886)
θ_L	0.00324 (0.00089, 0.00569)	0.00340 (0.00099, 0.00591)	0.00344 (0.00065, 0.00615)	0.00366 (0.00088, 0.00633)
θ_R	0.00458 (0.00264, 0.00664)	0.00443 (0.00252, 0.00646)	0.00475 (0.00283, 0.00679)	0.00447 (0.00265, 0.00642)
θ_D	0.01473 (0.00500, 0.03006)	0.01377 (0.00476, 0.02885)	0.02535 (0.00756, 0.04667)	0.02289 (0.00688, 0.04267)
θ_E	0.02275 (0.00800, 0.04140)	0.02174 (0.00741, 0.03986)	0.02252 (0.00786, 0.04429)	0.02061 (0.00710, 0.03971)
θ_{DH}	0.00257 (0.00028, 0.00743)	0.00254 (0.00028, 0.00734)	0.00319 (0.00030, 0.00936)	0.00326 (0.00029, 0.00953)
θ_{EH}	0.00603 (0.00030, 0.01639)	0.00727 (0.00030, 0.01828)	0.00655 (0.00030, 0.01893)	0.00763 (0.00032, 0.02029)

	Full data, model A, one rate	Full data, model A, Dir (2)	Full data, model C, one rate	Full data, model C, Dir (2)
φ	0.49210 (0.28676, 0.68465)	0.46917 (0.27794, 0.67775)	0.53450 (0.35757, 0.70527)	0.53371 (0.35866, 0.70634)
τ_R	0.00114 (0.00090, 0.00139)	0.00109 (0.00087, 0.00132)	0.00120 (0.00099, 0.00142)	0.00112 (0.00092, 0.00133)
τ_D	0.00032 (0.00020, 0.00047)	0.00030 (0.00019, 0.00043)	$= \tau_H$	$= \tau_H$
τ_E	0.00025 (0.00004, 0.00040)	0.00028 (0.00004, 0.00041)	$= \tau_H$	$= \tau_H$
τ_H	0.00018 (0.00000, 0.00033)	0.00022 (0.00000, 0.00034)	0.00036 (0.00027, 0.00044)	0.00035 (0.00027, 0.00044)
θ_W	0.00586 (0.00397, 0.00785)	0.00593 (0.00404, 0.00797)	0.00669 (0.00474, 0.00874)	0.00667 (0.00480, 0.00867)
θ_P	0.02255 (0.00039, 0.04056)	0.02595 (0.00038, 0.04121)	0.02113 (0.01593, 0.02677)	0.02152 (0.01627, 0.02717)
θ_L	0.00961 (0.00353, 0.01406)	0.01055 (0.00419, 0.01459)	0.01164 (0.00884, 0.01445)	0.01183 (0.00905, 0.01475)
θ_R	0.00620 (0.00389, 0.00884)	0.00586 (0.00373, 0.00823)	0.00568 (0.00381, 0.00773)	0.00555 (0.00364, 0.00747)
θ_D	0.01806 (0.00816, 0.02998)	0.01577 (0.00727, 0.02620)	0.01289 (0.00710, 0.01970)	0.01206 (0.00664, 0.01837)
θ_E	0.03038 (0.01642, 0.04671)	0.02847 (0.01492, 0.04429)	0.02960 (0.01582, 0.04579)	0.02712 (0.01507, 0.04143)
θ_{DH}	0.01444 (0.00025, 0.05232)	0.00899 (0.00026, 0.04458)	NA	NA
θ_{EH}	0.00155 (0.00017, 0.00438)	0.00175 (0.00019, 0.00469)	NA	NA

Note.— "One rate" means that the same rate is assumed for all loci, while Dir(2) means that rates vary among loci according to a Dirichlet distribution with shape parameter α = 2.

Table S2. Posterior means (in bold) and 95% HPD intervals (below) of parameters under the MSci model for the Anopheles genomic data (see also table 1)

	θ_G	θ_C	θ_R	θ_L	θ_A	θ_Q	θ_o	θ_g	θ_a	θ_c	θ_d	θ_e	θ_b	θ_h	θ_f	τ_o	$\tau_g = \tau_h$	τ_a	τ_c	τ_d	$\tau_e = \tau_f$	τ_b	φ_h	φ_f
**2L1+2 coding**	**4.63**	**2.45**	**0.28**	**0.17**	**0.62**	**0.66**	**0.71**	**2.75**	**46.6**	**0.45**	**0.57**	**0.89**	**1.16**	**2.11**	**3.97**	**0.79**	**0.44**	**0.75**	**0.69**	**0.44**	**0.32**	**0.15**	**0.28**	**0.94**
	3.39	1.96	0.27	0.16	0.59	0.61	0.66	2.00	0.36	0.29	0.49	0.77	1.01	0.32	0.77	0.76	0.40	0.70	0.67	0.40	0.31	0.13	0.24	0.92
	6.02	2.99	0.30	0.19	0.66	0.70	0.76	3.54	215.0	0.62	0.69	1.00	1.30	5.56	10.24	0.82	0.47	0.80	0.71	0.47	0.33	0.16	0.32	0.96
**2L1+2 noncoding**	**8.64**	**4.91**	**0.74**	**0.31**	**0.96**	**4.83**	**1.35**	**0.46**	**1.87**	**1.17**	**1.54**	**1.24**	**1.61**	**0.68**	**3.40**	**1.49**	**0.26**	**1.39**	**1.28**	**0.88**	**0.58**	**0.27**	**0.00**	**0.98**
	7.13	4.29	0.70	0.30	0.93	3.60	1.30	0.43	0.57	0.52	1.45	1.17	1.52	0.64	0.58	1.47	0.24	1.29	1.25	0.86	0.57	0.25	0.00	0.97
	10.28	5.56	0.79	0.32	1.00	6.21	1.39	0.49	3.17	1.63	1.64	1.31	1.71	0.72	8.20	1.51	0.29	1.49	1.30	0.89	0.59	0.28	0.00	0.99
**2La coding**	**11.41**	**2.36**	**0.73**	**0.24**	**0.81**	**7.67**	**0.85**	**0.24**	**1.53**	**1.98**	**0.28**	**0.53**	**1.97**	**0.47**	**2.12**	**0.76**	**0.18**	**0.60**	**0.60**	**0.58**	**0.24**	**0.19**	**0.01**	**0.73**
	6.95	1.94	0.61	0.23	0.74	3.31	0.80	0.21	1.36	0.36	0.17	0.49	0.89	0.42	1.46	0.74	0.16	0.58	0.58	0.57	0.23	0.16	0.00	0.70
	16.98	2.79	0.87	0.26	0.89	13.94	0.89	0.27	1.72	4.73	0.37	0.59	3.09	0.52	2.87	0.77	0.20	0.61	0.61	0.59	0.25	0.22	0.01	0.77
**2La noncoding**	**19.62**	**3.37**	**1.10**	**0.41**	**1.22**	**8.91**	**1.14**	**0.46**	**2.22**	**3.03**	**2.11**	**0.73**	**1.49**	**0.65**	**2.91**	**1.69**	**0.38**	**1.14**	**1.14**	**1.14**	**0.42**	**0.39**	**0.00**	**0.64**
	15.53	3.11	1.03	0.39	1.16	6.17	1.10	0.43	2.12	0.39	0.37	0.70	0.30	0.61	2.56	1.67	0.35	1.13	1.13	1.13	0.41	0.37	0.00	0.63
	24.02	3.65	1.18	0.41	1.28	12.2	1.18	0.50	2.32	7.70	5.22	0.77	2.73	0.70	3.27	1.71	0.41	1.16	1.16	1.15	0.43	0.41	0.00	0.65
**2R coding**	**6.75**	**1.87**	**0.25**	**0.16**	**0.74**	**0.66**	**0.61**	**3.30**	**2.07**	**0.28**	**0.58**	**0.85**	**0.95**	**1.80**	**4.71**	**0.70**	**0.35**	**0.67**	**0.64**	**0.35**	**0.28**	**0.17**	**0.34**	**0.97**
	4.98	1.63	0.24	0.15	0.66	0.61	0.57	2.55	0.53	0.18	0.51	0.06	0.16	0.36	0.74	0.68	0.31	0.66	0.63	0.31	0.23	0.14	0.30	0.96
	8.78	2.13	0.27	0.17	0.81	0.71	0.64	4.05	5.14	0.38	0.64	1.79	1.41	4.28	12.87	0.74	0.38	0.69	0.65	0.38	0.33	0.22	0.39	0.98
**2R noncoding**	**9.85**	**3.32**	**0.59**	**0.33**	**1.25**	**1.37**	**0.66**	**7.51**	**1.24**	**1.61**	**0.92**	**1.57**	**1.83**	**2.07**	**12.75**	**1.84**	**0.90**	**1.36**	**1.32**	**0.90**	**0.53**	**0.30**	**0.22**	**0.97**
	8.69	3.13	0.55	0.32	1.21	1.25	0.07	2.82	0.88	0.20	0.80	1.44	1.71	0.35	1.56	1.52	0.75	1.26	1.26	0.75	0.52	0.29	0.12	0.96
	11.06	3.53	0.63	0.34	1.29	1.48	1.36	14.4	1.49	5.15	1.15	1.75	1.95	5.17	38.38	2.11	1.00	1.63	1.38	1.00	0.55	0.30	0.34	0.99
**3L1+2 coding**	**3.02**	**1.71**	**0.30**	**0.19**	**0.56**	**0.50**	**0.78**	**1.38**	**1.01**	**1.38**	**0.41**	**0.91**	**0.88**	**2.48**	**6.40**	**0.72**	**0.43**	**0.69**	**0.67**	**0.43**	**0.22**	**0.06**	**0.32**	**0.94**
	1.34	1.02	0.27	0.17	0.51	0.46	0.72	0.97	0.35	0.28	0.34	0.80	0.75	0.34	1.17	0.69	0.39	0.66	0.65	0.39	0.20	0.05	0.27	0.92
	5.35	2.53	0.32	0.20	0.61	0.54	0.84	1.83	2.06	3.57	0.48	1.02	1.00	5.53	15.92	0.75	0.45	0.72	0.70	0.45	0.23	0.07	0.37	0.96
**3L1+2 noncoding**	**7.63**	**2.70**	**0.58**	**0.36**	**0.95**	**0.89**	**1.51**	**5.64**	**1.58**	**0.61**	**0.69**	**1.60**	**1.67**	**2.10**	**8.11**	**1.37**	**0.84**	**1.32**	**1.28**	**0.84**	**0.39**	**0.13**	**0.33**	**0.96**
	4.90	2.21	0.56	0.34	0.90	0.86	1.45	4.32	0.53	0.33	0.63	1.51	1.54	0.33	1.90	1.35	0.82	1.28	1.25	0.82	0.38	0.11	0.30	0.95
	10.99	3.23	0.60	0.37	1.00	0.93	1.57	7.07	3.18	0.89	0.75	1.69	1.80	5.30	17.93	1.40	0.86	1.37	1.30	0.87	0.40	0.14	0.36	0.97
**3La coding**	**14.96**	**6.68**	**0.40**	**0.20**	**1.07**	**1.16**	**0.84**	**0.78**	**1.66**	**1.73**	**0.53**	**1.64**	**1.71**	**1.99**	**5.92**	**0.68**	**0.38**	**0.67**	**0.67**	**0.38**	**0.21**	**0.13**	**0.65**	**0.93**
	4.95	3.43	0.38	0.19	0.95	1.06	0.79	0.67	0.32	0.32	0.46	1.39	0.98	0.37	1.26	0.66	0.36	0.64	0.63	0.36	0.20	0.10	0.62	0.91
	31.55	11.03	0.41	0.22	1.20	1.26	0.88	0.89	4.06	4.23	0.59	1.91	2.49	4.92	14.15	0.70	0.39	0.70	0.70	0.39	0.23	0.16	0.68	0.95
**3La noncoding**	**189.3**	**16.95**	**0.94**	**0.38**	**1.56**	**1.91**	**0.48**	**1.54**	**2.64**	**1.88**	**0.86**	**2.07**	**3.87**	**2.13**	**3.73**	**2.05**	**0.93**	**1.46**	**1.45**	**0.93**	**0.46**	**0.24**	**0.54**	**0.98**
	39.12	11.80	0.81	0.35	1.47	1.67	0.01	0.98	0.44	0.29	0.69	1.71	3.08	0.37	0.73	1.61	0.65	1.37	1.37	0.65	0.43	0.22	0.46	0.97
	634.5	22.84	1.03	0.42	1.66	2.07	1.44	2.49	5.23	5.27	1.12	2.74	4.59	5.25	9.00	2.27	1.10	1.61	1.60	1.10	0.48	0.27	0.70	0.98
**3R coding**	**8.32**	**4.14**	**0.31**	**0.20**	**0.84**	**0.81**	**0.76**	**1.97**	**2.92**	**0.37**	**0.85**	**4.78**	**0.69**	**1.98**	**6.40**	**0.74**	**0.32**	**0.71**	**0.67**	**0.32**	**0.25**	**0.17**	**0.43**	**0.95**
	5.54	3.22	0.28	0.17	0.75	0.71	0.72	1.48	0.83	0.25	0.68	1.11	0.17	0.34	1.17	0.72	0.26	0.68	0.66	0.26	0.21	0.13	0.37	0.93
	11.66	5.13	0.33	0.22	0.93	0.91	0.80	2.44	6.60	0.48	1.01	12.91	1.31	4.90	16.89	0.77	0.39	0.73	0.68	0.39	0.29	0.21	0.49	0.96
**3R noncoding**	**15.27**	**6.68**	**0.87**	**0.36**	**1.48**	**4.08**	**0.92**	**0.56**	**1.99**	**0.39**	**1.75**	**1.59**	**2.20**	**0.55**	**6.12**	**1.61**	**0.51**	**1.31**	**1.30**	**0.82**	**0.56**	**0.25**	**0.03**	**0.98**
	12.35	5.96	0.79	0.35	1.43	2.20	0.06	0.46	1.09	0.10	1.63	1.51	2.09	0.21	0.76	1.38	0.38	1.27	1.25	0.81	0.55	0.24	0.01	0.97
	18.49	7.43	0.93	0.38	1.52	5.56	1.41	0.71	3.01	0.70	1.90	1.68	2.31	0.75	16.46	2.02	0.73	1.38	1.37	0.83	0.57	0.26	0.06	0.98

Note.— Estimates of θs and τs are multiplied by 100.
